# Sarcolemma resilience and skeletal muscle health require *O*-mannosylation of dystroglycan

**DOI:** 10.1186/s13395-024-00370-2

**Published:** 2025-01-09

**Authors:** Jeffrey M. Hord, Sarah Burns, Tobias Willer, Matthew M. Goddeeris, David Venzke, Kevin P. Campbell

**Affiliations:** Department of Molecular Physiology and Biophysics, and Department of Neurology, Howard Hughes Medical Institute, Senator Paul D. Wellstone Muscular Dystrophy Specialized Research Center, Roy J. and Lucille A. Carver College of Medicine, The University of Iowa, Iowa City, Iowa USA

**Keywords:** Dystroglycan, *O*-mannosylation, Protein *O*-mannosyltransferase 1 (POMT1), Like-acetylglucosaminyltransferase 1 (LARGE1), Matriglycan, Skeletal muscle

## Abstract

**Background:**

Maintaining the connection between skeletal muscle fibers and the surrounding basement membrane is essential for muscle function. Dystroglycan (DG) serves as a basement membrane extracellular matrix (ECM) receptor in many cells, and is also expressed in the outward-facing membrane, or sarcolemma, of skeletal muscle fibers. DG is a transmembrane protein comprised of two subunits: alpha-DG (α-DG), which resides in the peripheral membrane, and beta-DG (β-DG), which spans the membrane to intracellular regions. Extensive post-translational processing and *O*-mannosylation are required for α-DG to bind ECM proteins, which is mediated by a glycan structure known as matriglycan. *O*-mannose glycan biosynthesis is initiated by the protein *O*-mannosyltransferase 1 (POMT1) and POMT2 enzyme complex and leads to three subtypes of glycans called core M1, M2, and M3. The lengthy core M3 is capped with matriglycan. Genetic defects in post-translational *O*-mannosylation of DG interfere with its receptor function and result in muscular dystrophy with central nervous system and skeletal muscle pathophysiology.

**Methods:**

To evaluate how the loss of *O*-mannosylated DG in skeletal muscle affects the development and progression of myopathology, we generated and characterized mice in which the *Pomt1* gene was specifically deleted in skeletal muscle (Pomt1^skm^) to interfere with POMT1/2 enzyme activity. To investigate whether matriglycan is the primary core M glycan structure that provides the stabilizing link between the sarcolemma and ECM, we generated mice that retained cores M1, M2, and M3, but lacked matriglycan (conditional deletion of *like-acetylglucosaminyltransferase 1*; Large1^skm^). Next, we restored *Pomt1* using gene transfer via AAV2/9-MCK-mPOMT1 and determined the effect on Pomt1^skm^ pathophysiology.

**Results:**

Our data showed that in Pomt1^skm^ mice *O*-mannosylated DG is required for sarcolemma resilience, remodeling of muscle fibers and muscle tissue, and neuromuscular function. Notably, we observed similar body size limitations, sarcolemma weakness, and neuromuscular weakness in Large1^skm^ mice that only lacked matriglycan. Furthermore, our data indicate that genetic rescue of *Pomt1* in Pomt1^skm^ mice limits contraction-induced sarcolemma damage and skeletal muscle pathology.

**Conclusions:**

Collectively, our data indicate that DG modification by Pomt1/2 results in core M3 capped with matriglycan, and that this is required to reinforce the sarcolemma and enable skeletal muscle health and neuromuscular strength.

**Supplementary Information:**

The online version contains supplementary material available at 10.1186/s13395-024-00370-2.

## Background

Skeletal muscle development and function requires linkage between the extracellular matrix (ECM) in the basement membrane and the skeletal muscle fiber membrane (i.e., sarcolemma). Dystroglycan (DG) is an extensively glycosylated transmembrane receptor that links the ECM to the sarcolemma [1]. The DG protein is comprised of two interacting subunits: alpha-DG (α-DG) and beta-DG (β-DG). α-DG is the peripheral membrane subunit in the basement membrane that interacts with laminin globular (LG) domain-containing ligands, such as laminin, agrin, and perlecan [1]. β-DG is a transmembrane subunit that creates a link to the intracellular cytoskeleton by binding to sub-sarcolemmal dystrophin and utrophin [1]. Thus, DG acts as a bridge between the extracellular matrix (ECM) and the intracellular cytoskeleton.

**Fig. 1:**
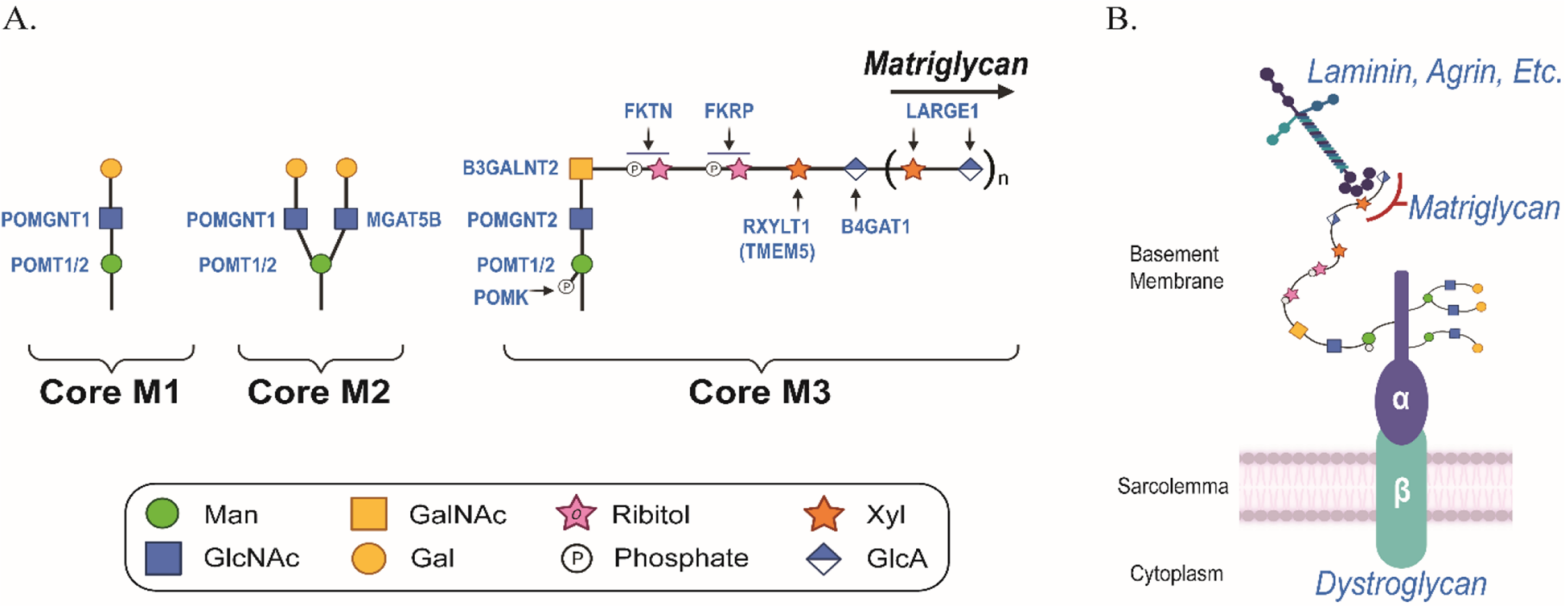
Biosynthesis of core M glycans on α-DG. **A** The α-DG *O*-mannosyl glycosylation pathway is depicted, highlighting the synthesis of core M1, M2, and M3 glycans. **B** Schematic depicts *O*-mannosylated DG situated at the sarcolemma and its interaction with ECM ligands. Abbreviations: Man, mannose; GlcNAc, *N*-acetylglucosamine; Gal, galactose; GalNAc, *N*-acetylgalactosamine; Xyl, xylose; GlcA, glucuronic acid; POMT1/2, protein *O*-mannosyltransferases 1 and 2; POMGNT1, protein *O*-linked mannose *N*-acetyl-glucosaminyltransferase 1; MGAT5B, mannosyl α1,6-glycoprotein β1,6,-*N*-acetyl-glucosaminyltransferase; POMGNT2, protein *O*-linked mannose *N*-acetyl-glucosaminyltransferase 2; B3GALNT2, β1,3-*N*-acetylgalactosaminyltransferase 2; POMK, protein *O*-mannose kinase; FKTN, Fukutin; FKRP, Fukutin related protein; RXYLT1, ribitol xylosyltransferase 1; TMEM5, transmembrane protein 5; B4GAT1, β1,4-glucuronyltransferase 1; LARGE1, like-acetyl-glucosaminyltransferase 1

Binding between DG and the ECM requires the addition of *O*-mannosyl glycans to the mucin-like domain in the N-terminal region of α-DG [2–4]. To date, at least 18 genes have been reported to be directly or indirectly involved in *O*-mannosylation of α-DG [5, 6]. *O*-mannose glycan biosynthesis is initiated in the endoplasmic reticulum by the protein *O*-mannosyltransferase 1 (POMT1) and protein *O*-mannosyltransferase 2 (POMT2) enzyme complex (Fig. 1A). The POMT1/2 complex catalyzes the transfer of dolichol phosphate-activated mannose to the hydroxyl oxygen of serine or threonine side chains on the α-DG mucin domain [7]. Further modifications building on the initial *O*-mannose results in elongated glycan structures called core M1, M2, and M3 [6]. Synthesis of the core M1 glycan results from addition of β1,2-linked *N*-acetylglucosamine (GlcNAc) to the *O*-mannose by protein *O*-linked mannose *N*-acetylglucosaminyltransferase 1. Core M1 glycans can undergo additional extension and can serve as a precursor for the core M2 glycan. The core M2 glycans are formed by adding β1,6-linked GlcNAc to a core M1 though the activity of mannosyl α1,6-glycoprotein β1,6-*N*-acetyl-glucosaminyltransferase (MGAT5B). Multiple core M2 glycan structures exist. Biosynthesis of the core M3 glycan begins with the addition of β1,4-linked GlcNAc to the *O*-mannose by POMGNT2. Extension proceeds with the addition of β1,3-linked *N*-acetylgalactosamine to the GlcNAc by β1,3-*N*-acetylgalactosaminyltransferase 2. Once the trisaccharide has been assembled within the endoplasmic reticulum (ER), protein *O*-mannose kinase phosphorylates the carbon six-position of the *O*-mannose. Further extension of the phosphorylated core M3 occurs within the Golgi complex. Tandem ribitol-1-phosphate additions are catalyzed by Fukutin and Fukutin-related protein. The ribitol is modified with β1,2-xylose (Xyl) by ribitol xylosyltransferase 1 (RXYLT1; formerly known as transmembrane protein 5), followed by transfer of β1,4-linked glucuronic acid to the Xyl by β1,4-glucuronyltransferase1 (B4GAT1). RXYLT1 and B4GAT1 modifications serve as a primer for extension by like acetyl-glucosaminyltransferase 1 (LARGE1), which then polymerizes an α1,3-Xyl-β1,3-GlcA repeat. The sequential actions of the glycosyltransferases and phosphor-kinase result in the heteropolysaccharide called matriglycan [6] (Fig. 1A), which binds with high affinity to LG-domain-containing ECM proteins (Fig. 1B). Although the functions of the core M1 and M2 have not been deciphered, substantial progress has been made in understanding the importance of the core M3 glycan.

Defects in the enzymes involved in *O*-mannosyl processing of α-DG that impair its function result in a group of muscular dystrophies known as dystroglycanopathies [2, 8]. Specifically, mutations in the POMT1 gene result in limb-girdle muscular dystrophy (LGMD R11 POMT-1 related) [9–13] and Walker Warburg Syndrome (WWS) [14], which presents with brain and eye abnormalities. Intriguing in vitro findings suggest that the specific location of *O-*mannosylation by POMT1/2 influences where alternative serine and threonine *N*-acetylgalactosamine modifications occur on α-DG [15]. Therefore, the absence of *O*-mannosylation may result in aberrant *O*-GalNAc modifications on α-DG, which could harbor disease-modifying effects in vivo. Unfortunately, genetic disruption of *Pomt1* in mice results in early embryonic lethality [16], thereby creating a barrier to discern the mechanisms and phenotypes of POMT1 dystroglycanopathies. Recently, our research group and others have established POMT1 conditional knockout mice, which has furthered our understanding of how POMT1 is involved in retinal photoreceptor ribbon synapses [17] and cardiac muscle [18]; however, our understanding of the mechanisms by which POMT1 contributes to skeletal muscle health and thus prevents pathophysiology remains unclear.

In the current study, we describe the generation of a mouse model in which POMT1 is specifically deleted in skeletal muscle (Pomt1^skm^). Pomt1^skm^ mice exhibited sarcolemma instability, aberrant muscle remodeling, skeletal muscle dysfunction, runted body size, and premature death. Notably, using a LARGE1 skeletal muscle-specific knockout (Large1^skm^) mouse line, we provide evidence suggesting that core M3 capped with matriglycan is the critical core M glycan structure that provides sarcolemma reinforcement and promotes skeletal muscle health. Lastly, our data demonstrate that disease progression in Pomt1^skm^ was halted when *Pomt1* expression was restored through gene replacement.

## Methods

### Animals

Animal care, ethical usage, and procedures were performed in strict accordance with protocols approved by the National Institutes of Health and the Institutional Animal Care Use and Committee (IACUC). Mice were socially housed (unless single housing was required), under specific-pathogen-free conditions in an Association for Assessment and Accreditation of Laboratory Animal Care (AAALAC)-accredited animal facility. Mouse housing conditions were as specified in the Guide for the Care and Use of Laboratory Animals (National Research Council). A reverse 12 h/12 h light/dark cycle was used, and in vivo mouse assessments only took place during the dark cycle. Standard rodent chow (Harlan Laboratories, Indianapolis, IN, USA) and water were available ad libitum. *Pomt1*^loxP/loxP^ and *Large1*^loxP/loxP^ mice were used as controls. When available, littermates were used as controls. Both male and female mice were used. Within each experiment, mice were age-matched and sex-matched when possible. Group designations (randomization) were assigned based on identification numbers and genotype information before the experimenter observed the mice to exclude any bias based on mouse phenotype. Due to the progressive deterioration in motor and respiratory function with age, target mice (Pomt1^skm^ and Large1^skm^) were euthanized due to reduced body score, inability to feed, hindlimb paralysis, or respiratory distress, as dictated in our IACUC protocol. Animal usage and data reporting were in accordance with the Animal Research: Reporting of In Vivo Experiments (ARRIVE) guidelines.

### Generation of Pomt1^skm ^mice

To generate a skeletal muscle-specific *Pomt1* conditional knockout mouse model, we designed a breeding strategy using the Cre/LoxP system to obtain target mice carrying homozygous *Pomt1* floxed alleles and the paired box 7 (Pax7)-Cre transgene (*Pomt1*^loxP/loxP^/*Pax7*^Cre^) (Supplemental Fig. 1A and B). Please refer to Hord et al. [18] for detailed description of our floxed *Pomt1* (*Pomt1*^loxP/loxP^) mice. The *Pax7*^Cre^ transgene drives Cre recombinase activity off of the *Pax7* gene promoter, which is expressed in early skeletal muscle development and proliferation (embryonic day 7) and in satellite cells initiating muscle regeneration [19]. Transgenic *Pax7*^Cre^ mice (*Pax7*^*tm1(cre)Mrc*^*/J*: Strain #010530; RRID:IMSR_JAX:010530) were purchased from The Jackson Laboratory (Bar Harbor, ME, USA).

*Pomt1*^loxP/loxP^ mice were crossed with mice expressing *Pax7*^Cre^ to produce mice heterozygous for the *Pomt1* floxed allele and the *Pax7*^Cre^ transgene (*Pomt1*^loxP/+^/*Pax7*^Cre^). To generate *Pomt1*^loxP/loxP^/*Pax7*^Cre^ target mice, male *Pomt1*^loxP/+^/*Pax7*^Cre^ mice were bred with female *Pomt1*^loxP/loxP^ mice. All mice were subjected to genotyping either by PCR analysis or outsourced to Transnetyx (Cordova, TN, USA). In skeletal muscle tissues, exons 3 and 4 of *Pomt1* were expected to be deleted in both *Pomt1* alleles by Cre-driven recombination, thus generating *Pomt1* null alleles. To assess intragenic *Pomt1* deletion in mouse skeletal muscle, genomic DNA, muscle was extracted, and DNA was isolated. Primers, *Pomt1*-Forward primer 5’-CCA CCC AGC ACT TAA CCT TTT A-3’ and *Pomt1*-Reverse primer 5’-ACT GTA TAT GCC TGG CCA CTG T-3’ yielded a 223 bp product for wild-type allele and a 203 bp band for the *Pomt1*-floxed allele. *Pax7*^Cre^ transgene expression was performed as instructed by The Jackson Laboratory using the transgene forward primer (oIMR1084) 5’-GCG GTC TGG CAG TAA AAA CTA TC-3’ and transgene reverse primer (oIMR1085) 5’-GTG AAA CAG CAT TGC TGT CAC TT-3’ to yield a 100 bp product.

### Generation of Large1^skm^ mice

*Large1*^loxP/loxP^ mice were crossed with *Pax7*^Cre^ mice to produce mice that were heterozygous for the *Large1* floxed allele and the *Pax7*^Cre^ transgene (*Large1*^loxP/+^/*Pax7*^Cre^). Please refer to Hord et al. [18] for detailed description of our floxed *Large1* mice. Male *Large1*^loxP/+^/*Pax7*^Cre^ mice were bred with female *Large1*^loxP/loxP^ mice to achieve *Large1*^loxP/loxP^/*Pax7*^Cre^ mice. Every mouse was subjected to genotyping either by PCR analysis or outsourced to Transnetyx. In skeletal muscle tissues, exon 3 of *Large1* was expected to be deleted in both *Large1* alleles by Cre-driven recombination, thus generating *Large1* null alleles. To assess intragenic *Large1* deletion in genomic DNA from mouse skeletal muscle, muscle was extracted, and DNA was isolated. Primers, *Large1* – Forward primer 5’-TGG CAT TGT GGC AGG TAA CAG-3’ and *Large1* – Reverse primer 5’-TCC ACA CAT GGT ATG TAC TCA CT-3’ yielded a 383 bp product for the wild-type allele and a 444 bp band for the *Large1*-floxed allele.

### Large^myd^/Large^myd^ mice

The *Large*^*myd*^*/Large*^*myd*^ mice were used as a natural model of *Large1*-deficient muscular dystrophy [2, 20]. *Large*^*myd*^*/Large*^*myd*^ mice (The Jackson Laboratory, stock #000300; MYD/Le-*Os* + / + *Large*^*myd*^/J) were maintained on a C57BL/6 J background, and colony maintenance was carried out by laboratory staff.

### Mouse phenotyping

To evaluate the health of skeletal muscle in our experimental mice, in vivo and ex vivo assays were used to assess the functionality of the skeletal muscle and neuromuscular systems.

#### Grip testing

The forelimb grip strength experimental protocol was performed in line with the procedures described in de Greef et al. [21] except where stated. Briefly, mice were permitted to acclimate to the procedure room for 10 min. A mouse grip strength meter (Columbus Instruments, Columbus, OH, USA) was mounted horizontally, with a nonflexible grid connected to the force transducer. Mice performed five sets of three pulls of the grid with a 1 min break between each set. The mean of the three highest pulls of all fifteen pulls was used to determine the average mouse grip strength.

#### Treadmill

The mice were acclimated to the treadmill (AccuPacer Treadmill, Omnitech Electronics, Inc., Columbus, OH, USA) the day before the exercise protocol. Acclimation consisted of mice being placed on the stationary treadmill (0 m per minute (mpm)) for 20 min at a 15° decline with the shock grates activated. Exercise consisted of a progressive downhill running protocol (15° decline; 3 mpm for 5 min – 7.5 mpm for 3 min – 10 mpm for 3 min – 12.5 mpm for 3 min – 15 mpm for 3 min). Mice that remained on the shock grates for 10-s or more were removed from the treadmill.

#### Serum creatine kinase

Blood was collected by tail vein bleeds from non-anesthetized, restrained mice using a scalpel blade and a Microvette CB300 (Sarstedt AG and Co, Newton, NC, USA) as previously described [21, 22]. Samples were prepared in accordance with the manufacturer’s instructions using an enzyme-coupled CK kit (Stanbio Laboratory, Boerne, TX, USA).

#### Hanging test

Balance, motor coordination, and skeletal muscle condition were assessed with the hanging test. The wire hang test was conducted as previously described [23] except where noted. Briefly, a 2 mm thick metal rod was secured approximately 37 cm above a thick layer of mouse cage bedding. Mice were lifted by their tail and brought within reaching distance of the wire. The timer was started once the mouse grasped the wire with their forepaws and the experimenter released the tail. Improper behaviors such as balancing on top of the wire or deliberately jumping off the wire were addressed by replacing the mouse on the wire without stopping the timer. A fixed hanging time limit of 180 s was used. As soon as the mouse fell off the wire, the timer was stopped, and the time was recorded. Mice that were able to hang for 180 s were gently removed from the wire and returned to their cage. The maximum hanging time out three trials was used for further analysis.

#### Open-field voluntary activity

Open-field voluntary locomotor activity was measured using a DigiScan apparatus (Omnitech Electronics) as previously described [24]. Following three days of acclimation, mouse activity data was collected for two consecutive days, and the higher of the two values for each reported parameter was used for analysis.

#### In vivo plantar flexion torque

Plantar flexion of the triceps surae was measured using an in vivo muscle contractility apparatus (Model 1300A, Aurora Scientific, Inc., Aurora, Ontario, Canada). Mice were anesthetized via inhaled isoflurane delivered at 3–4% for induction and 1.5–2.5% for maintenance anesthesia (SomnoSuite Low-Flow Anesthesia System, Kent Scientific, Torrington, CT, USA). Ophthalmic lubricating gel was applied to both eyes to prevent dryness and corneal irritation. Mice were placed in a supine position on a thermostatically-controlled testing platform set at 37 °C. The right knee was secured using blunt clamps and the right foot was firmly fixed onto the footplate that was connected to the servomotor. The hind paw was taped to the force plate and positioned so that the foot and the tibia were aligned at approximately 90°. Platinum electrodes were placed subcutaneously in proximity to the tibial nerve for stimulation of the plantar flexor muscles. Optimal isometric twitch torque was determined by increasing the current with a minimum of 30-s between each twitch contraction to avoid fatigue. Static performance was determined through an isometric tetanic contraction at 100 Hz and 125 Hz for 200-ms each with the ankle set at 90° (angle between the tibia and foot). Each isometric contraction was followed by a 2 min rest to avoid fatigue. Dynamic eccentric contractions were then performed. Eccentric contractions consisted of a 200-ms isometric contraction followed by a lengthening contraction in which the footplate traversed 30° at a velocity of 40°/s. The muscles were then passively returned to resting length. The susceptibility to contraction-induced force loss was examined via 6 eccentric contractions with 30-s pauses between each contraction. For experiments assessing muscle fiber damage, 20 eccentric contractions were performed. Maximal force of the isometric plateau (occurring during the 200-ms prior to eccentric stretch) was measured and used to normalize the decrease in force production.

#### Ex vivo isolated EDL muscle force

Ex vivo skeletal muscle contractile properties were assessed in extensor digitorum longus (EDL) muscles that were surgically removed and analyzed as previously described [24, 25]. Briefly, the muscle was immediately placed in a bath containing a physiological salt solution bubbled with 95% O_2_ and 5% CO_2_ to stabilize the pH at 7.4 and maintained at 25 °C. The distal tendon was tied to a dual-mode servomotor (Model 1200A Isolated Muscle Test System for Mice; Aurora Scientific, Inc.) and the proximal tendon was clamped to the post. Following optimization steps, the maximal isometric tetanic force was determined. Next, the muscle was subjected to an eccentric contraction (ECC) protocol consisting of five ECCs separated by 3 min rest intervals. Each ECC consisted of an initial 100-ms isometric contraction at optimal frequency immediately followed by a stretch of optimal muscle length to 30% of fiber length beyond optimal length, and then the muscle was passively returned to optimal length. After the analysis of EDL contractile properties, the muscle was weighed. The cross-sectional area (CSA) of the EDL was determined by dividing the muscle mass by the product of fiber length and the density of mammalian skeletal muscle (1.06 g/cm^3^). The specific force was determined by dividing maximal isometric force by the CSA (mN/mm^2^).

### Antibodies

The following primary antibodies have been described previously and were obtained from the listed sources: IIH6 monoclonal antibody [26] to detect matriglycan (Campbell Laboratory; Developmental Studies Hybridoma Bank, University of Iowa; RRID:AB_2617216); recombinant rabbit IIH6 antibody (provided by Amicus Therapeutics); MANDA G2 7D11 [27] to detect β-DG (Campbell Laboratory); affinity purified β-DG rabbit polyclonal AP83 [28] (Campbell Laboratory); AF6868 rabbit polyclonal antibody to detect core α-DG and β-DG proteins (Campbell Laboratory; R and D Systems, Minneapolis, MN; RRID:AB_10891298); monoclonal anti-perlecan (A7L6; Invitrogen, Waltham, MA); neurofilament H (NF-H; EnCor; CPCA-NF-H); synaptophysin (Thermo Fisher Scientific; MA5-14,532); and polyclonal anti-caveolin-3 (ab2912; Abcam). Secondary antibodies conjugated to Alexa Fluor 488, Alexa Fluor 555, Alexa Fluor 594, and Alexa Fluor 647 were purchased from Invitrogen.

### Histology and immunofluorescence

Mice were euthanized by cervical dislocation and skeletal muscle tissues were immediately harvested. Skeletal muscle tendons were trimmed, blood was removed by wicking, and wet weights were obtained. Muscles were embedded in freezing medium (Tissue-Tek O.C.T. compound; Sakura FineTek; Torrance, CA, USA) and immediately snap frozen in liquid nitrogen-cooled isopentane (2-methylbutane). Ten μm sections were cut with a cryostat (Leica CM3050S Research Cryostat; Amsterdam, the Netherlands) set at -20ºC. Cryosections were processed for hematoxylin and eosin (H&E) staining according to standard protocols [26]. Picrosirius red (0.1%) & Fast Green (0.1%) staining was performed on skeletal muscle cryosections to evaluate fibrotic accumulation [21]. For immunofluorescence, cryosections were blocked with Background Buster (NB306; Innovex Biosciences, Richmond, CA, USA), incubated in primary antibodies overnight, washed in PBS, followed by incubation in secondary antibodies (1:500), washed in PBS, and cover-slipped with mounting medium containing the nuclear marker DAPI (ProLong Gold Antifade Mountant with DAPI; Invitrogen). In select cases, anti-mouse IgG (1:250; Invitrogen) was used to detect myofiber damage via immune-cell infiltration and was added along with secondary antibodies. Digital images were acquired with either the VS120-S5-FL slide scanner microscope (Olympus Corporation, Tokyo, Japan) or the FLUOVIEW FV3000 confocal laser scanning microscope (Olympus). Quantitative analysis was performed using VS-Desktop software (Olympus) and cellSens analysis software (Olympus).

NMJ assessment was performed as previously described [25]. EDL and transverse abdominis (TVA) were harvested and immediately washed in PBS three times for 5 min each. Then, muscles were fixed in 4% paraformaldehyde for 20 min followed by three washes in PBS. Fixed EDL muscle samples were split into three to four fiber bundles, and TVA muscles remained intact, before incubating in 3% Triton X-100/PBS for 3 h at 4 °C. Muscles were subsequently washed in PBS followed by blocking at 4 °C for 4 h in Background Buster (Innovex; NB306). Samples incubated in primary antibodies against NFH and synaptophysin at 4 °C overnight. The muscles were then washed in PBS and incubated with fluorescently conjugated secondary antibodies and Alexa Fluor 488-conjugated α-bungarotoxin (Invitrogen; B13422) for 2 h. Images were acquired using an Olympus FLUOVIEW FV3000 confocal laser scanning microscope. Complete enface NMJs were identified and acquired with Z-stacks using 20x, 60x, and 100 × objectives. Maximum intensity Z-stacks were reconstructed with the FV31S (Olympus) software and deconvoluted with cellSens Dimension (Olympus).

### Glycoprotein enrichment and immunoblotting

Skeletal muscle tissues were minced and then placed in Tris-buffered saline (TBS) containing 1% Triton-X-100 and protease inhibitors for processing [29]. The solubilized fraction was combined with a WGA-agarose bead slurry (Vector Laboratories, Burlingame, CA) and incubated overnight at 4ºC with rotation. Pellets formed from the beads and were subsequently washed three times in 0.1% Triton X-100/TBS [29]. The beads were eluted with 5X Laemmli sample buffer (LSB) at 99ºC for 10 min. Samples were separated by 3–15% SDS-PAGE and transferred to polyvinylidene fluoride (PVDF)-FL membranes. The membranes were incubated with primary antibodies followed by the appropriate infrared (IR) dye-conjugated secondary antibodies. Immunoblots were scanned using the Odyssey infrared imaging system (LI-COR Bioscience, Lincoln, NE, USA) and images were subsequently captured with the Odyssey image analysis software (LI-COR Bioscience).

### LARGE enzymatic activity

For assessment of endogenous LARGE GlcA-T activity in skeletal muscle, quadricep muscles were solubilized in 250 µl of 50 mM Tris–HCl, pH 7.4, 100 mM NaCl, 1% TX-100, with protease inhibitors (0.6 µg/ml of Pepstatin A, 0.5 µg/ml of leupeptin with aprotinin, 0.75 mM benzamidine, and 1 mM phenylmethanesulfonyl fluoride). The samples were subsequently spun down at 30,000 × g for 30 min. The HPLC-based LARGE enzymatic assay was performed as previously described [30, 31]. Skeletal muscle supernatants (20 µl) were incubated in 50 µl of 0.4 mM Xyl-1,3-GlcA-MU and 10 mM UDP-GlcA in 0.1 M MES buffer, pH 6.0, 5 mM MnCl2, and 5 mM MgCl2 for 18 h at 37 °C. The reaction was terminated by adding 0.1 M EDTA and boiling for 5 min. The supernatants were analyzed using a 4.6 × 250 mm Supelcosil LC-18 column (Supelco, Belefonte, PA, USA) with Buffer A (50 mM ammonium formate, pH 4.0) and Buffer B (80% acetonitrile in Buffer A), using a 9% B isocratic run at 1 ml/min using HPLC (Shimadzu Scientific, Kyoto, Japan). The elution of MU derivatives was monitored by fluorescence detection (325 nm for excitation and 380 nm for emission) and peak area was used as a measure of activity. The GlcA-T activity was assessed by subtracting the background observed in the negative control sample without donor sugar and normalized against the amount of protein measured using the DC protein assay (Bio-Rad, Hercules, CA, USA).

### AAV vector production and in vivo administration

AAV vector production and injection procedures were performed as previously described [18]. The sequence encoding mouse *Pomt1* was synthesized (Genscript, Piscataway, NJ, USA) and then cloned into the adeno-associated virus (AAV) backbone under the transcriptional control of the striated muscle-specific creatine kinase (*MCK*) promoter (a gift from Jeffrey Chamberlain at the University of Washington, Seattle). The AAV2/9 vector contains the genome of serotype 2 packaged in the capsid from serotype 9 and was selected due to its ability to improve muscle transduction efficiency as well as alter tropism. The vector AAV2/9-MCK-Pomt1 was generated by the University of Iowa Viral Vector Core Facility. Pomt1^skm^ mice (4 weeks old) received a single, 50 μL injection of the vector solution (3.28 × 10^13^ vg/ml) via the retro-orbital sinus intravenous route.

### Statistics

All data in the present study are shown as the means ± standard deviation unless noted otherwise. The number of sampled units, *n*, is a single mouse for an experiment (i.e., one mouse is *n* = 1). GraphPad Prism 9 software was used for all statistical analyses. Two-tailed t-test was used when a single data set was compared to a separate data set. Multiple unpaired t-test with Holm-Sidak post-hoc test was used for age-matched comparisons. Ordinary One-Way ANOVA with Tukey’s method for multiple comparisons. Differences were considered significant at a *p*-value less than 0.05.

## Results

### Core M glycans are required on α-DG in skeletal muscle fibers for muscle health and survival

**Fig. 2:**
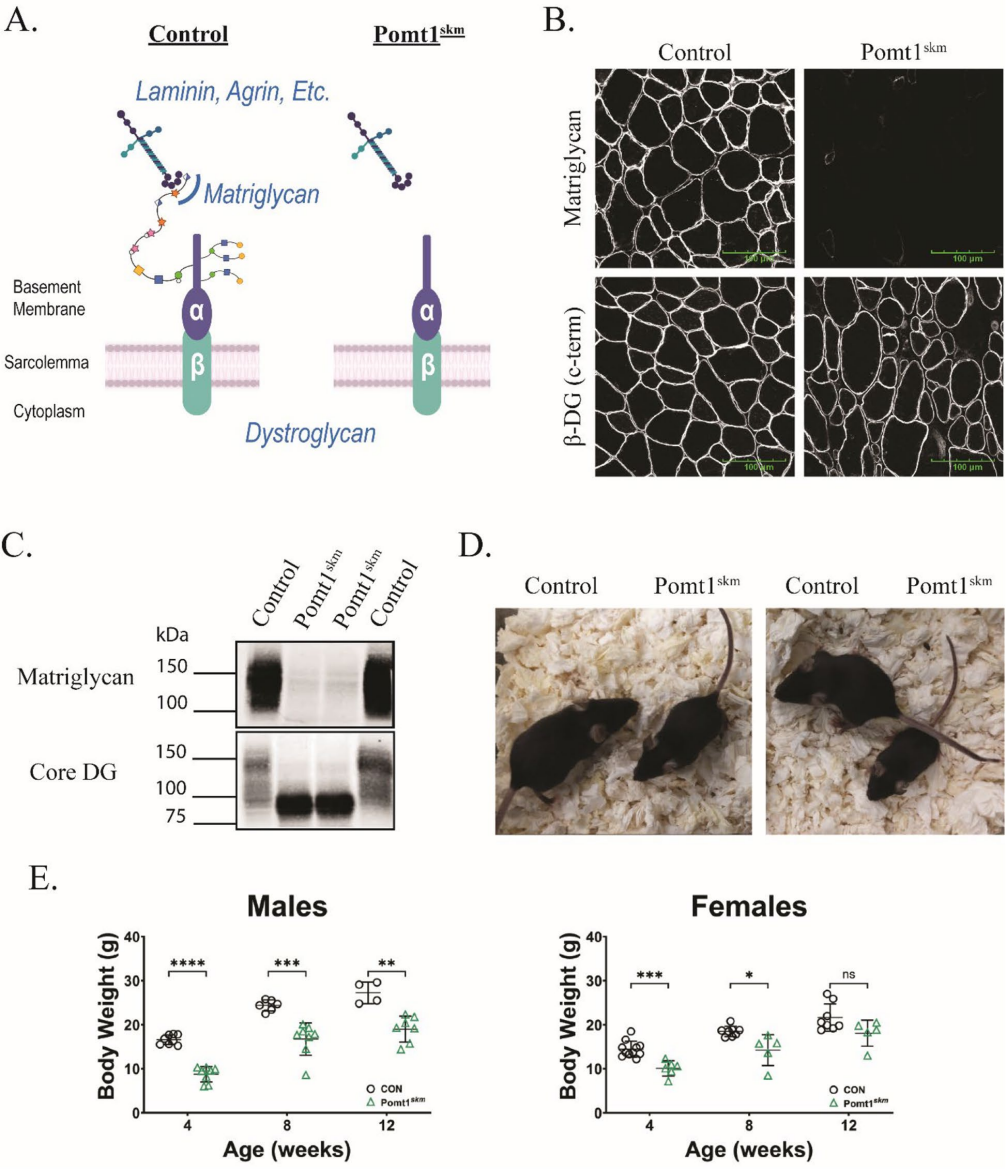
Successful production of skeletal muscle-specific Pomt1-deficient mice. **A** Schematic depicting *O-*mannosylated dystroglycan in the sarcolemma of control and Pomt1-deficient skeletal muscle (Pomt1^skm^). **B** Immunofluorescence for matriglycan (recombinant rabbit IIH6 antibody) and the C-terminus of β-DG (rabbit polyclonal AP83 antibody) in transverse cross-sections of the gastrocnemius muscle from control or Pomt1^skm^ mice. Images captured at 40X magnification. Scale bar = 100 µm. **C** Western blot analysis of pooled skeletal muscle samples from 13-week-old female control and Pomt1^skm^ mice. The monoclonal IIH6 antibody was used to detect matriglycan and the AF6868 rabbit polyclonal antibody was used to detect core DG. **D** Pictures of two Pomt1^skm^ mice alongside littermate controls. All four mice were 4 weeks of age. **E** Body weights of Pomt1.^skm^ and age- / gender-matched controls at 4-, 8-, and 12 weeks of age. Data are expressed as mean ± standard deviation. P-values determined by unpaired t-tests with Holm-Sidak post-hoc analysis. Males: ** = 0.0011; *** = 0.0008; **** < 0.00001. Females: ns, not significant; * = 0.0161; *** = 0.0007

We successfully generated mice with skeletal muscle-specific deletion of POMT1 (Pomt1^skm^) using Cre/loxP breeding strategies (described in the *Methods* and Supplemental Fig. 1A and B). The frequency of Pomt1^skm^ target mice fell within the expected range of allelic distribution (Supplemental Fig. 1C). The lack of POMT1in the skeletal muscle of Pomt1^skm^ mice led to a DG protein that lacked *O*-mannosylation within its mucin domain (Fig. 2A). As there is currently no reliable antibody to detect POMT1 protein in mammalian tissues, we indirectly assessed POMT1 expression via the detection of matriglycan. Immunofluorescent analysis of skeletal muscle obtained from 13-week-old mice revealed that matriglycan was absent in Pomt1^skm^ muscle, whereas it was detected in skeletal muscle from control mice (Fig. 2B). β-DG was observed along the sarcolemma in sections from both control and Pomt1^skm^ mice (Fig. 2B). Western blot analysis using wheat-germ agglutinin (WGA)-enriched extracts of skeletal muscle pooled from control mice or Pomt1^skm^ mice also showed that matriglycan was absent in Pomt1^skm^ muscle (Fig. 2C). However, the presence of core α-DG protein was detected in muscle from both control and Pomt1^skm^ mice, although the blot from the Pomt1^skm^ muscle lacked the characteristic vertical elongation indicative of the extensive glycosylation of α-DG (Fig. 2C).

Pomt1^skm^ mice were visibly smaller at four weeks of age (Fig. 2D), and their body weights were significantly lower than those of age- and gender-matched controls at four, eight, and 12 weeks of age, although the difference was not significant between 12-week-old females (Fig. 2E). Deaths occurred as early as four weeks of age in Pomt1^skm^ mice; by eight weeks, a quarter of all Pomt1^skm^ mice had died (Supplemental Fig. 1D). Together, these findings indicate that Pomt1^skm^ mice are viable and lack matriglycan.

**Fig. 3:**
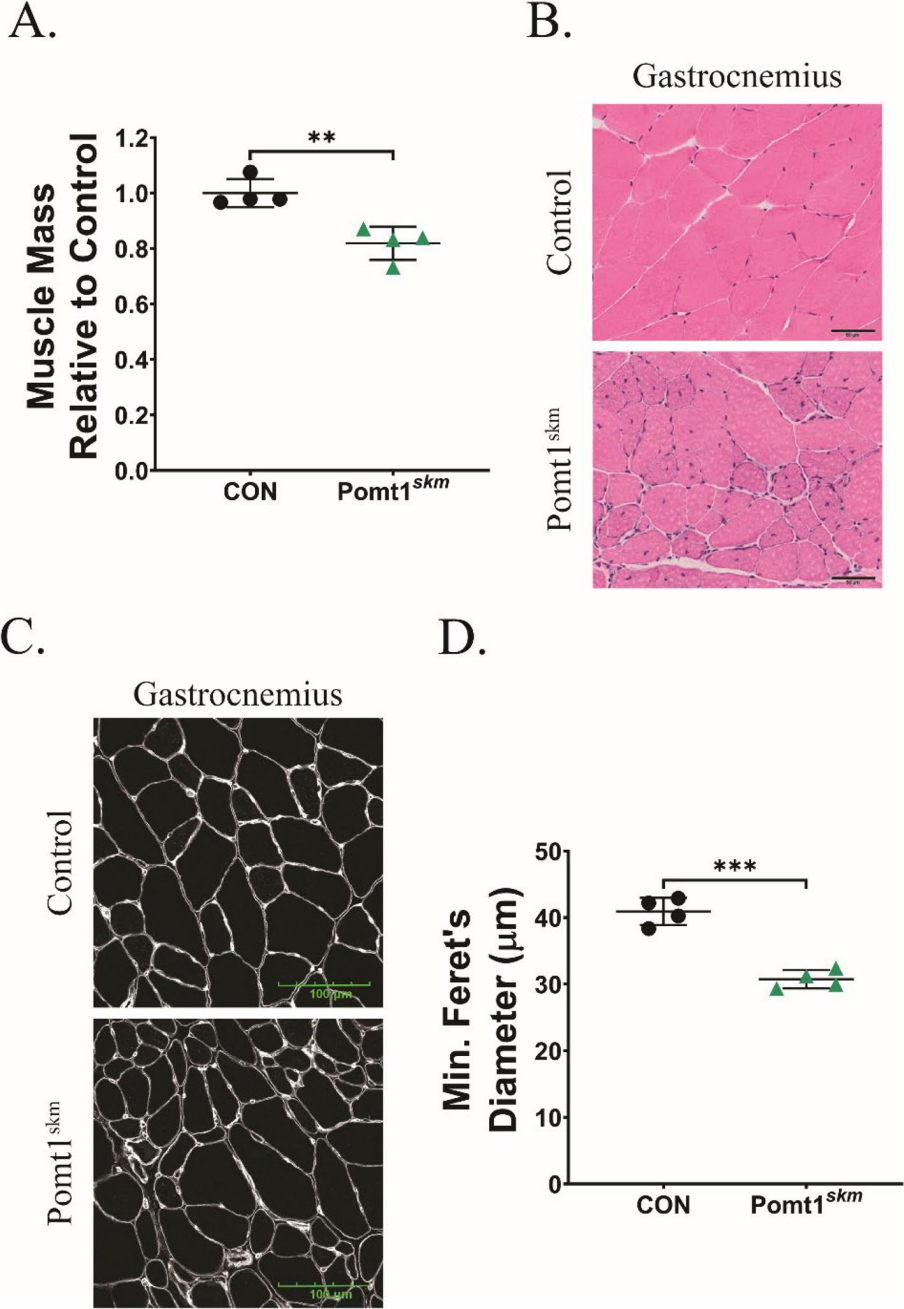
Skeletal muscle mass and fiber diameter are influenced by *O*-mannosylation of α-DG. **A** Skeletal muscle mass for gastrocnemius (Gast) muscle tissue from 13-week-old male control mice and age- and gender-matched Pomt1^skm^ mice. N = 4 / group. **B** Hematoxylin and eosin (H&E) stained transverse cross-sections from gastrocnemius muscles obtained from control or Pomt1^skm^ mice. Images captured with 20X objective. Scale bar = 50 µm. **C** and **D** Anti-perlecan-stained immunofluorescent images obtained from transverse cross-sections of the mid-belly of gastrocnemius muscles from control and Pomt1.^skm^ mice (C). Minimal (min) Feret’s diameter determined from anti-perlecan images (D). N = 4 per group. Data expressed as mean ± standard deviation. P-values determined by unpaired t-test with Holm-Sidak post-hoc analysis. Gast * = 0.0037. Min. Feret’s Diameter *** = 0.0002

We next assessed how the loss of POMT1 in skeletal muscle affected muscle weight and size. The wet weights of skeletal muscle in gastrocnemius and gluteus muscles were significantly lower in Pomt1^skm^ mice than those in age- and gender-matched controls (Fig. 3A; Supplemental Fig. 2A). Histological analysis of the quadriceps, gastrocnemius, gluteus maximus, and diaphragm revealed dystrophic pathology, including variable fiber size, inflammatory cell infiltration, and internal nuclei (Fig. 3B; Supplemental Fig. 2B). In addition, the minimal Feret’s diameter of Pomt1^skm^ gastrocnemius muscle fibers averaged 10.6 µm smaller than the diameter of control fibers (41.2 vs. 30.6 µm) (Fig. 3C and D). Thus, the loss of POMT1 in skeletal muscle diminishes muscle mass and fiber diameter.

### Neuromuscular function requires core M glycans on α-DG in skeletal muscle

**Fig. 4:**
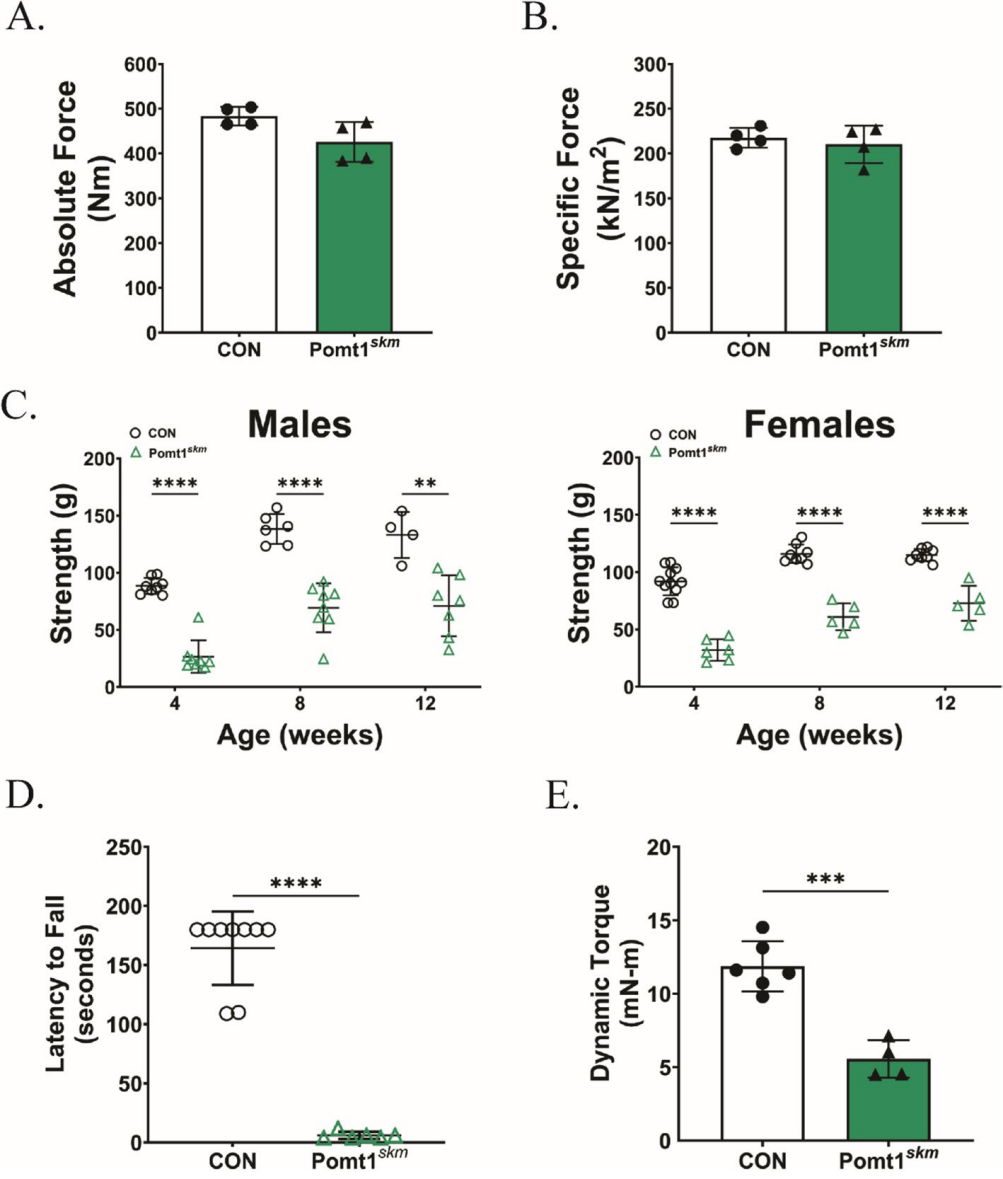
POMT1 is required in skeletal muscle for neuromuscular function. **A** and **B** Ex vivo maximum isometric tetanic contractions of the extensor digitorum longus (EDL) from control and Pomt1^skm^ mice to detect A) absolute force and B) specific force indicating absolute force relative to the estimated cross-sectional area of the EDL muscle N = 4 per group. **C** Neuromuscular strength assessed through forelimb grip strength testing in control mice and age- and gender-matched Pomt1^skm^ mice at four, eight, and 12 weeks of age. Males: ** = 0.0030; **** < 0.0001. Females: **** < 0.0001. **D** Neuromotor coordination and skeletal muscle endurance assessed through wire hang experiment. **** < 0.0001. **E** Plantar flexor torque produced during dynamic movement from in vivo electrical stimulation. *** = 0.0002. For all statistical analyses, unpaired t-tests with Holm-Sidak post-hoc analysis were performed. Data expressed as mean ± standard deviation

We next assessed how the absence of POMT1 in skeletal muscle affected muscle function and neuromuscular health. We excised extensor digitorum longus (EDL) muscles from control and Pomt1^skm^ mice for ex vivo examination of their contractile properties (Fig. 4A and B). Maximal isometric tetanic contractile force and force per EDL cross-sectional area (i.e., specific force) were not significantly lower in Pomt1^skm^ muscle compared to age- and gender-matched controls (Fig. 4A and B). Ex vivo contractile force is a measure of non-voluntary muscle contraction but does not capture the functional strength of skeletal muscle that involves voluntary neuromuscular effort. Therefore, we proceeded to assess neuromuscular function through a variety of in vivo tests. In vivo analysis of forelimb grip force at 4-, 8-, and 12 weeks of age revealed that grip strength was significantly lower in Pomt1^skm^ mice compared to age- and gender-matched controls (Fig. 4C). We also assessed neuromuscular strength, endurance, and coordination using the wire hang assay. The majority of control mice were able to remain on the wire apparatus for the allotted 180-s whereas Pomt1^skm^ mice were unable to remain on the wire (Fig. 4D). In vivo analysis of the plantar flexors revealed that Pomt1^skm^ mice produced significantly lower torque output than those of control mice (Fig. 4E). Thus, Pomt1 is required for neuromuscular strength.

We next assessed voluntary locomotor activity at baseline and post-exercise conditions in control and Pomt1^skm^ mice. In an open field activity assay, Pomt1^skm^ mice traveled a shorter distance, had fewer vertical activity events (i.e., rearing and/or hopping), and spent less time performing vertical events at baseline and post-exercise (Supplemental Fig. 3A and B). Further evidence of neuromotor disruption was observed when Pomt1^skm^ mice were suspended from their tails and displayed hindlimb clasping (Supplemental Fig. 3C), a characteristic often associated with neuromotor diseases [32–34]. Due to the accumulating evidence of neuromuscular dysfunction in Pomt1^skm^ mice, we next evaluated neuromuscular junctions in four-week-old mice. Compared to control mice, the transversus abdominis muscle from Pomt1^skm^ mice exhibited abnormal post-synaptic morphology while remaining innervated (Supplemental Fig. 3D). Overall, these data suggest that *O*-mannosylation of α-DG influences neuromuscular strength and performance, potentially by disrupting post-synaptic function.

### Core M glycans on α-DG reinforce the sarcolemma and are critical for muscle fiber remodeling

**Fig. 5:**
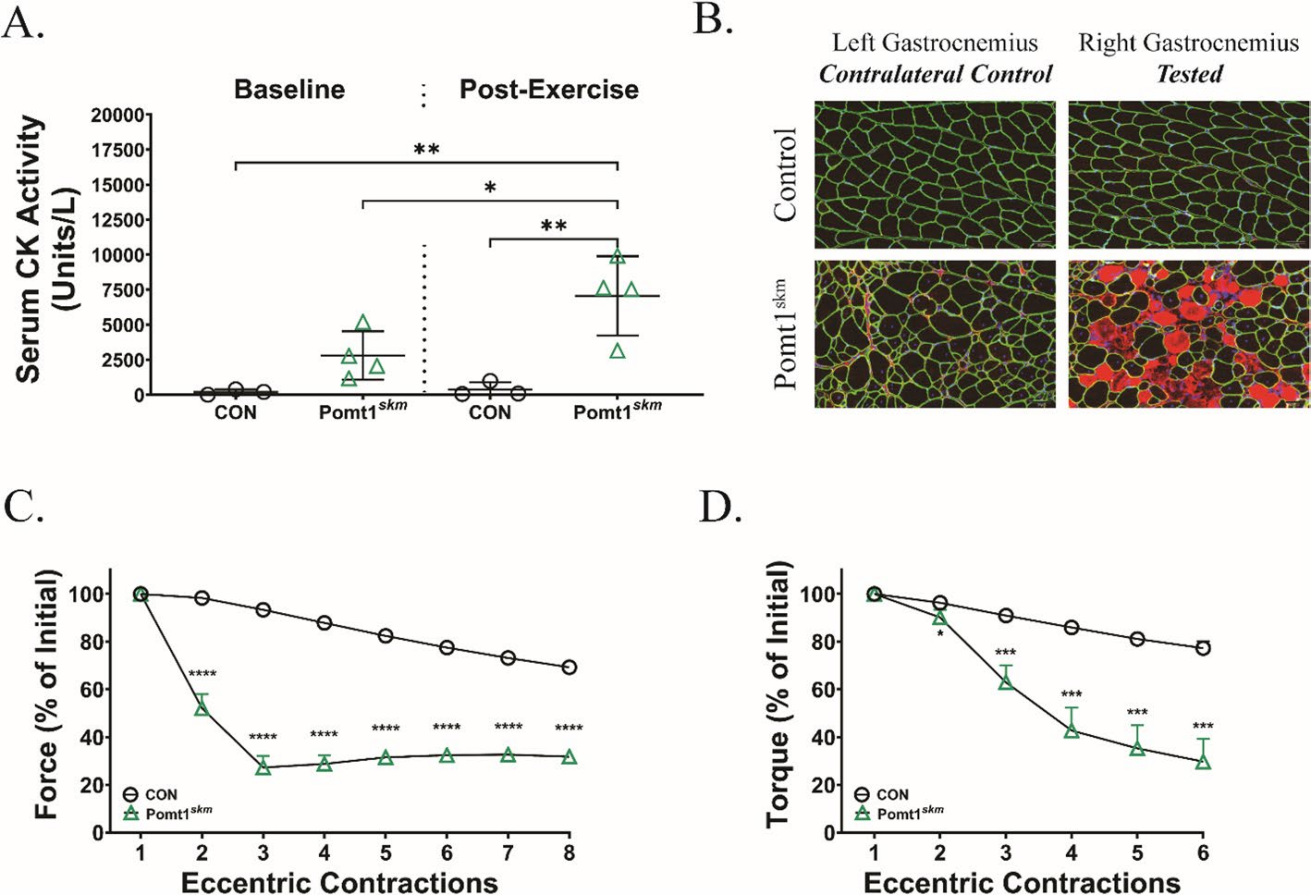
Sarcolemma strength relies on *O*-mannosylation of α-DG. **A** Serum creatine kinase (CK) activity was evaluated in control and Pomt1^skm^ mice to indirectly assess the extent of sarcolemma damage under baseline conditions and two hours after a downhill treadmill running exercise. Data expressed as mean ± standard deviation. P-values determined by ordinary one-way ANOVA with Tukey’s multiple comparisons test. * = 0.0355; ** < 0.004. **B** Immunofluorescence of cross-sections of gastrocnemius muscles harvested three days after control or Pomt1^skm^ mice endured a single bout of isometric and forty eccentric contractions. Muscle from the left leg was used as a contralateral control for the muscle tested on the right leg. Sections were stained with antibodies to detect β-DG (rabbit polyclonal AP83; green), mouse IgG (orange), and DAPI (blue). Images shown at 20X magnification. **C** Percent of initial force in repeated ex vivo lengthening contractions in extensor digitorum longus (EDL) muscles control (CON) or Pomt1^skm^ mice. N = 4 / group. Data expressed as mean ± standard deviation. P-values determined by unpaired t-test with Holm-Sidak post-hoc analysis. **** < 0.0005. **D** In vivo assessment of plantar flexor response, as measured by the torque in response to repeated eccentric contractions in CON or Pomt1.^skm^ mice. Data expressed as mean ± standard deviation. N = 5 / group. Data expressed as mean ± standard deviation. P-values determined by unpaired t-test with Holm-Sidak post-hoc analysis. * < 0.05; *** < 0.001

Another potential source of skeletal muscle dysfunction in Pomt1^skm^ mice is plasma membrane weakness. Thus, we investigated whether repeated muscle contractions are required to exacerbate the skeletal muscle phenotype. Creatine kinase (CK) is a striated muscle protein that escapes into circulation upon damage or injury to the muscle cell membrane (sarcolemma). We first assessed the presence of serum CK under baseline conditions and two hours after a downhill treadmill running exercise. Under baseline conditions, serum CK was slightly, but not significantly, higher in Pomt1^skm^ mice relative to controls (~ 2,700 vs. ~ 100 Units/L; Fig. 5A). However, 2 h after a single bout of progressive downhill treadmill exercise, Pomt1^skm^ mice had a significant increase in serum CK (~ 7,000 Units/L) compared to controls (baseline and post-exercise) and baseline Pomt1^skm^ levels. At the same time, control mice showed only a modest increase in serum CK activity following treadmill running, remaining below 500 Units/L.

Next, we harvested the gastrocnemius/plantaris complex two days after mice endured 40 eccentric muscle contractions. Pomt1^skm^ muscles displayed patches of IgG-positive muscle fibers, indicating that those muscle fibers had been damaged (Fig. 5B). To determine if sarcolemma fragility led to loss of force production, we isolated EDL muscles and assessed their susceptibility to repeated stressors ex vivo. Although Pomt1^skm^ EDL muscles produced similar levels of isometric force relative to controls (Fig. 4A and B), Pomt1^skm^ muscles were unable to maintain force production in the presence of repeated lengthening contractions, whereas controls could maintain this force production (Fig. 5C). Similarly, Pomt1^skm^ plantar flexors were highly susceptible to repeated eccentric contraction-induced damage (Fig. 5D). Our findings reveal that Pomt1^skm^ skeletal muscle fibers are highly susceptible to even mild, sustained muscle stress, indicating that *O*-mannosylation of α-DG is required for sarcolemma strength.

Based on the extent of sarcolemma fragility observed in the Pomt1^skm^ mice, we next sought to determine how the absence of POMT1 affected skeletal muscle remodeling. Internal and central localized nuclei are an indication of skeletal muscle fiber remodeling. We found that under baseline conditions, Pomt1^skm^ mice have numerous observable central nucleated fibers (CNF) in both the gastrocnemius and gluteus maximus muscles compared to muscles from control mice (Supplemental Fig. 4A and B). Quantification of CNF in the gastrocnemius revealed that 40.3% of Pomt1^skm^ fibers had central nuclei, whereas only 0.88% of fibers in the gastrocnemius of control mice had central nuclei (Supplemental Fig. 4B). In addition to an elevated percentage of CNF in Pomt1^skm^ skeletal muscle, other indicators of skeletal muscle remodeling were evident, such as noticeable increases in fibers expressing embryonic myosin heavy chain (eMHC; Supplemental Fig. 4C), abnormal clustering of post-synaptic acetylcholine receptors and morphology of neuromuscular junctions (Supplemental Fig. 4D), disorganization of the microtubule network (Supplemental Fig. 4E), and fibrosis throughout skeletal muscle tissues (Supplemental Fig. 4F). These findings indicate that *O*-mannosylation of α-DG is a key factor in the remodeling process within skeletal muscle.

### Core M3 glycans and matriglycan are critical for sarcolemma stability

**Fig. 6:**
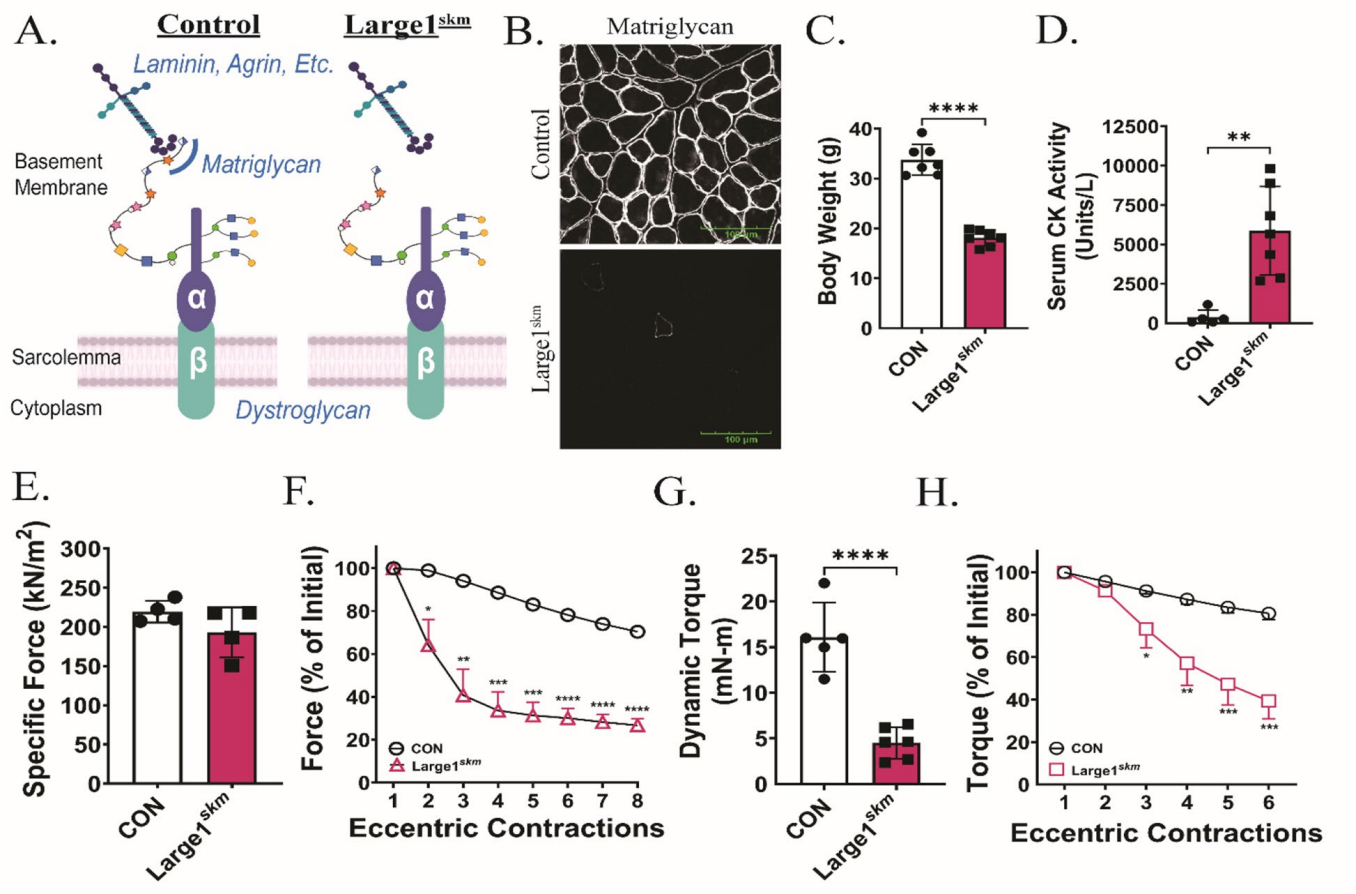
Skeletal muscle health requires matriglycan. **A** Schematic of DG core M glycans in control and Large1^skm^ mice. **B** Immunofluorescence of skeletal muscle to detect matriglycan (recombinant rabbit IIH6 antibody) in control and Large1^skm^ mice. Images shown at 40X magnification. Scale bar = 100 µm. **C** Body weights of 20-week-old control and Large1^skm^ mice. **** < 0.0001. **D** Serum creatine kinase (CK) activity two hours after treadmill exercise. ** = 0.0016. **E** Specific force determined by ex vivo maximum isometric tetanic contractions of the EDL muscle. **F** Ex vivo analysis of force in EDL muscles following eccentric contractions. N = 4 per group. **G** In vivo dynamic torque production by plantar flexors. **** < 0.0001. **H** Isometric torque as a measure of the response of plantar flexors to repeated eccentric contractions. N = 5 CON; N = 6 Large1^skm^. Data expressed as mean ± standard deviation. P-values determined by unpaired t-test with Holm-Sidak post-hoc analysis

To address whether the core M3 glycan modification on DG is essential for maintaining skeletal muscle membrane integrity, we generated mice in which the *Large1* (like-acetylglucosaminyltransferase 1) gene was deleted in skeletal muscle (Large1^*skm*^) using Cre/loxP breeding strategies (described in the *Methods* and Supplemental Fig. 5A and B). The LARGE1 protein is the final glycosyltransferase involved in forming core M3 glycan structures and is responsible for generating matriglycan (Fig. 1A and B; Fig. 6A). Thus, Large1^skm^ lack only the matriglycan cap on core M3 glycans and retain core M1 and M2 glycan structures (Fig. 6A). The frequency of Large1^skm^ target mice fell within the expected range of allelic distribution (Supplemental Fig. 5C). Immunofluorescent analysis revealed that matriglycan was absent from Large1^skm^ skeletal muscle fibers (Fig. 6B), while β-DG and dystrophin were localized within or near the sarcolemma (Supplemental Fig. 5D). Next, we examined LARGE1 enzymatic activity through the glucuronyltransferase (GlcA-T) activity assay. Large1^skm^ skeletal muscles were found to have only 7.69% LARGE1 activity compared to that of controls, whereas LARGE activity was not detected in the *Large*^*myd*^*/Large*^*myd*^ mice (Supplemental Fig. 5E), which harbor a spontaneous mutation in the *Large1* gene [2, 20]. Body weights of 16-week-old Large1^skm^ mice were significantly lower than control littermates (Fig. 6C). We next performed a series of experiments to determine how the loss of LARGE1 affects skeletal muscle function. Two hours after a downhill treadmill exercise, we observed that serum CK was significantly elevated compared to controls (Fig. 6D). Similar to the performance of Pomt1^skm^ EDL muscles, EDL muscles from Large1^skm^ mice produced a specific force comparable to that produced by control mice (Fig. 6E). However, Large1^skm^ mice were highly susceptible to loss of force induced by repeated eccentric contractions (Fig. 6F). In addition, the neuromuscular strength of plantar flexors was significantly lower in Large1^skm^ mice compared to controls (Fig. 6G), and repeated eccentric contractions led to a drop in torque production (Fig. 6H). Similar to the Pomt1^skm^ mice, deaths occurred as early as four weeks of age in the Large1^skm^ mice; by 16-weeks, 26.3% of all Large1^skm^ mice had died (Supplemental Fig. 5F). Our findings revealed similarities in the skeletal muscle phenotype observed between Large1^skm^ and Pomt1^skm^. This suggests that core M3 glycans containing matriglycan are the glycan structures required for maintaining skeletal muscle health and neuromuscular function.

### POMT1 gene transfer limits disease progression in Pomt1^skm^ mice

**Fig. 7:**
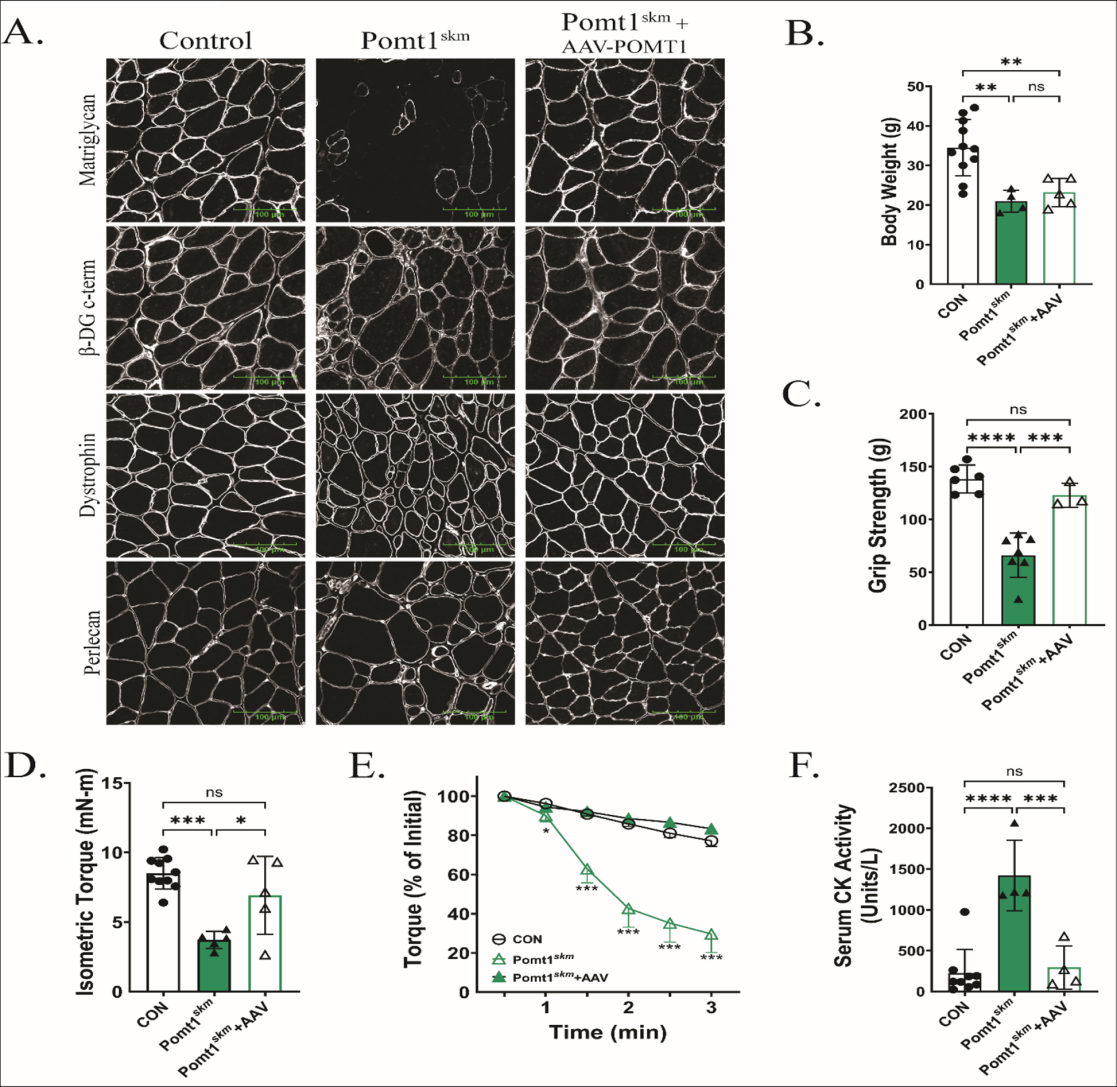
Restoring expression of *Pomt1* in Pomt1^skm^ mice limits skeletal muscle pathology. Four-week-old Pomt1^skm^ mice were administered AAV2/9-MCK-mPOMT1 (AAV-POMT1) via retro-orbital intravenous injection and effects were compared to control or Pomt1^skm^ mice. **A** Immunofluorescence for matriglycan (recombinant rabbit IIH6 antibody), the c-terminus of β-DG (MANDA G2 7D11 antibody), dystrophin, and perlecan in transverse cross-sections of gastrocnemius muscles. Images shown at 40X magnification. Scale bar = 100 µm. **B** Body weights of mice sixteen-weeks after AAV injection. ** < 0.007. **C** Forelimb grip strength four-weeks after AAV-POMT1 injection. *** = 0.0009; **** < 0.0001. **D-F**. Sixteen-weeks after AAV-POMT1 injection, in vivo plantar flexor torque production was assessed. D. isometric torque production with the ankle positioned at 90-degrees. * = 0.0158; *** = 0.0001. E. Response of plantar flexors to repeated eccentric contractions. * < 0.05; *** < 0.001. N = 8 CON; 5 Pomt1^skm^; 5 Pomt1^skm^ + AAV. F. Serum creatine kinase (CK) activity under baseline conditions to indirectly assess the extent of sarcolemma damage. *** = 0.0006; **** < 0.0001. P-values for all data determined by one-way ANOVA with Tukey’s multiple comparisons test. Data are expressed as mean ± standard deviation

Given our findings that the loss of POMT1 in skeletal muscle disrupts neuromuscular function, increases muscle fiber damage, and impairs muscle remodeling, we set out to determine if the gene transfer of *Pomt1* could halt or prevent disease progression. Therefore, we aimed to halt or prevent disease progression in Pomt1^skm^ mice by forcing *Pomt1* expression by administering adeno-associated virus (AAV)-*Pomt1*. We designed an AAV construct using serotype 2/9 and the muscle creatine kinase (*MCK*) promoter to drive expression of murine *Pomt1* in skeletal muscle tissues: AAV2/9-MCK-mPOMT1, hereafter referred to as AAV-POMT1. Male and female Pomt1^skm^ mice received AAV-POMT1 at four weeks of age via retro-orbital injection. Thirty weeks after AAV-POMT1 injection, skeletal muscles were analyzed for expression of matriglycan, β-DG, dystrophin, and perlecan. Immunofluorescent staining of Pomt1^skm^ mice in which POMT1 was expressed revealed robust matriglycan expression, as well as β-DG and dystrophin, all of which were localized near the sarcolemma (Fig. 7A). Sixteen weeks post-injection, body weights were no different in Pomt1^skm^ + AAV-POMT1 compared to untreated Pomt1^skm^ mice, and both groups weighed significantly less than controls (Fig. 7B). We next determined if restoring *Pomt1* expression had an effect on muscle function. Four weeks post-injection, Pomt1^skm^ + AAV-POMT1 mice had significantly higher grip strength than age-matched untreated Pomt1^skm^ mice (Fig. 7C). Moreover, 16 weeks post-AAV treatment, Pomt1^skm^ + AAV-POMT1 mice could produce significantly greater torque than Pomt1^skm^ during in vivo plantar flexor testing (Fig. 7D). Notably, AAV-POMT1 treatment led to a control-like response to eccentric contractions in plantar flexor muscles 16 weeks post-AAV treatment (Fig. 7E). Moreover, baseline serum CK in Pomt1^skm^ + AAV-POMT1 mice was similar to that in control mice, providing further evidence of improved sarcolemma strength following AAV-POMT1 treatment (Fig. 7F). Collectively, these observations indicate that the myopathy observed in Pomt1^skm^ mice can be limited or slowed by restoring *Pomt1* expression, and that this likely occurs through *O*-mannosylated DG providing strength to the sarcolemma.

## Discussion

The early embryonic lethality of mice deficient for *Pomt1* [16] underscores the essential role of *O*-mannosylation of α-DG during development. The use of Cre/LoxP technology to delete *Pomt1* or *Pomt2* in a time and tissue-specific manner has enabled a further understanding of the importance of *O*-mannosylated α-DG in various tissues, including the mouse brain [35], retina [17], and cardiac muscle [18]. Given the severe effect on skeletal muscle function in dystroglycanopathy patients with mutations in either POMT1 or POMT2, we developed a mouse model in which *Pomt1* was specifically deleted in skeletal muscle, which provides a mechanism to understand the role of *O*-mannosylated α-DG in skeletal muscle development and disease.

To assess the importance of *O*-mannosylation of α-DG for its interaction with the ECM during skeletal muscle development and maintenance, we bred floxed *Pomt1* mice to those expressing Cre recombinase under the control of the *Pax7* gene [19, 25]. This resulted in mice with a skeletal muscle-specific deletion of *Pomt1* (Pomt1^skm^) starting at embryonic day 7, in which *O*-mannosylation of α-DG was disrupted during skeletal muscle development, differentiation, and regeneration. In the current study, Pomt1^skm^ mice had a near complete absence of matriglycan in skeletal muscle tissues. They exhibited significant reductions in both body weight and select isolated muscle masses compared to age- and gender-matched littermates. Moreover, skeletal muscle from Pomt1^skm^ mice showed multiple signs of histopathology, including reduced muscle fiber diameter. These mice also displayed neuromuscular weakness, low levels of voluntary activity, and post-synaptic abnormalities. Notably, 25% of Pomt1^skm^ mice died before 8 weeks of age, and many of the Pomt1^skm^ mice died or required euthanasia before 35 weeks of age due to severe wasting, hind limb paralysis, or respiratory distress. In contrast, *Pomt1* gene deletion at embryonic day 17 using *MCK*-Cre resulted in mice whose lifespans could exceed 90 weeks [18]. Our current findings indicate that gene deletion starting at embryonic day 7 driven by *Pax7*-Cre led to a severe phenotype, with early onset of skeletal muscle pathology, reduced body size and weight, and a shortened lifespan, thus presenting a similar phenotype to that of dystroglycanopathy patients with mutations in *Pomt1*.

Our current data demonstrates that disrupting POMT1/2 activity results in physical impairment that can manifest or become exacerbated under conditions of muscle stress (i.e., forceful contractions, exercise, or increased physical activity). Following downhill treadmill exercise, Pomt1^skm^ mice had significantly elevated serum CK levels, indicative of sarcolemma damage, compared to their baseline levels and compared to the baseline and post-exercise levels of controls. Although Pomt1^skm^ EDL muscles produced isometric force that was similar to controls, our data indicate that repeated eccentric contractions resulted in precipitous loss of contractile function. A similar observation was noted in vivo following repeated eccentric contractions of the plantar flexor muscles of Pomt1^skm^ mice. In agreement with these data, our previous studies showed that lack of either DG, matriglycan, or the presence of shortened matriglycan renders skeletal muscle susceptible to eccentric contraction-induced strength loss [22, 24, 25, 36, 37].

We also observed an increase in IgG-positive fiber staining following in vivo eccentric contractions in Pomt1^skm^ mice, further confirming contraction-induced sarcolemma damage. This finding is similar to our previous observations in *Pomt1*-deficient hearts, in which acute pharmacological-induced contractile stress resulted in cardiac muscle fiber membrane damage [18]. The increased susceptibility to muscle damage that we observed in Pomt1^skm^ mice could result in attempts to remodel skeletal muscle fibers and muscle tissue. Accordingly, we observed evidence of remodeling and aberrant remodeling in Pomt1^skm^ mice, including increased central nucleation of muscle fibers, the presence of eMHC, aberrant morphology of NMJs, disorganized microtubule networks, and increased accumulation of muscle tissue fibrosis. Collectively, these data indicate that *O*-mannosylation of α-DG is required for sarcolemmal health and resiliency, and that its absence increases the susceptibility of contraction-induced muscle damage followed by ineffective remodeling of muscle tissue.

Skeletal muscle from Large1^skm^ lack only the matriglycan cap on core M3 glycans and retain core M1 and M2 glycan structures; thus, this model allowed us to determine if the core M3 glycan modification of α-DG is required to maintain the integrity of the sarcolemma. The similar pathophysiological findings observed in Pomt1^skm^ mice and Large1^skm^ mice suggest that matriglycan that is associated with core M3 provides a stabilizing link between the sarcolemma and ECM. Moreover, these data suggest that it is unlikely the core M1 and M2 glycans contribute to muscle development or stabilize the sarcolemma. These observations agree with our previous findings, which implicated core M3 plus matriglycan as the form of *O*-mannosylation on α-DG that provides structural integrity to cardiac muscle surface membranes [18]. Therefore, core M1 and core M2 glycans appear to be expendable in relation to striated muscle health and disease.

It is worth noting that we observed low levels of matriglycan in skeletal muscle from Pomt1^skm^ and Large1^skm^ mice. Based on our observations, matriglycan-positive skeletal muscle fibers were likely due to *Pomt1*- or *Large1*-deficient fibers undergoing degeneration followed by a regenerative process that escaped *Pax7*-Cre-driven deletion of the target gene. Over time, more and more knockout muscle fibers would be subjected to degeneration and regeneration, resulting in a growing number of revertant muscle fibers capable of producing matriglycan. Comparison of skeletal muscle from 13-week-old (Fig. 2B) and 34-week-old (Fig. 7A) Pomt1^skm^ shows an observable increase in matriglycan-positive fibers. Moreover, 21-week-old Large1^skm^ mice displayed low levels of LARGE activity rather than a complete absence of activity like that of the *Large*^*myd*^*/Large*^*myd*^ mice (Supplemental Fig. 5E). Although these data implicate revertant fibers as the source of matriglycan in conditional knockouts, it is also possible that *O-*mannosylation activity is occurring via alternative routes. For instance, recent discoveries of the functions of protein mannosyltransferases tetratricopeptide repeat-containing proteins 1–4 (TMTC1-4) [38] and transmembrane protein 260 (TMEM260) [39] open the possibility that compensatory *O*-mannosylation may occur in the absence of POMT1/2. The presence of LARGE activity and matriglycan in the absence of LARGE1 could be due to LARGE2 [31], although LARGE2 is predominately expressed in kidney and placental cells [40, 41]. Whether the presence of skeletal muscle matriglycan in the Pomt1^skm^ and Large1^skm^ mice was due to revertant fibers or compensatory glycosylation pathways is a topic for future investigations.

Our characterization of Pomt1^skm^ mice showed a phenotype that closely resembled that observed in humans with mutations in *Pomt1*. This indicates the utility of this model for therapeutic studies. Previously, our laboratory showed that adenovirus-mediated intramuscular *Large1* gene transfer into young *Large*^*myd/myd*^ mice prevented muscle pathology [42]. More recently, we demonstrated that systemic AAV-mediated gene transfer of *Large1* in adult *Large*^*myd/myd*^ mice improved the link between DG on skeletal muscle and the basement membrane; this was associated with improved health outcomes with no notable adverse effects [24]. We also recently showed that systemic administration of *Pomt1* with the AAV2/9 vector under the control of the *MCK* promoter effectively restored *Pomt1* expression in cardiac and skeletal muscle of *Pomt1*^loxP/loxP^; *MCK*^Cre^ striated muscle-specific knockout mice [18]. Similarly, systemically administering AAV2/9-MCK-POMT1 to Pomt1^skm^ mice restored biochemical defects, corrected histological abnormalities, enhanced neuromuscular strength, and improved resistance to eccentric stress in the Pomt1^skm^ mice. While these results indicate an improved phenotype in Pomt1^skm^ mice after *Pomt1* gene transfer, the treatment was unable to overcome developmental limitations that were already established, such as smaller body size. Nevertheless, our data indicate that the susceptibility and subsequent degenerative dystrophic process that occurs in Pomt1^skm^ mice can be limited or prevented by *Pomt1* gene transfer.

## Conclusions

Our findings demonstrate that the Pomt1^skm^ mouse model recapitulates skeletal muscle disease phenotypes, such as those observed in patients with dystroglycanopathies. Use of the *Pax7*-Cre driver allowed us to discriminate between myogenic and non-myogenic (neural, vascular, etc.) pathologic mechanisms in Pomt1^skm^ and Large1^skm^ mice. Furthermore, our study is the first to evaluate the beneficial effects of Pomt1 gene transfer under the control of a muscle-specific promoter on skeletal muscle. Thus, our findings underscore the essential influence that *O*-mannosylated α-DG has on the development and maintenance of skeletal muscle, and on the resiliency of skeletal muscle health.

## Supplementary Information

Supplementary Material 1.

## Data Availability

No datasets were generated or analysed during the current study.

## Abbreviations

AAV: Adeno-associated virus
AChRs: Acetylcholine receptors
B3GALNT2: β1,3-*N*-acetylgalactosaminyltransferase 2
B4GAT1: β1,4-glucuronyltransferase 1
CK: Creatine kinase
CNF: Central nucleated fibers
CON: Control
CSA: Cross-sectional area
DG: Dystroglycan
ECC: Eccentric contraction
ECM: Extracellular matrix
EDL: Extensor digitorum longus
eMHC: Embryonic myosin heavy chain
FKRP: Fukutin related protein
FKTN: Fukutin
Gal: Galactose
GalNAc: *N*-acetylgalactosamine
GalNAc: *N*-acetylgalactosamine
GAST: Gastrocnemius
GlcA: Glucuronic acid
GlcNAc: *N*-acetylglucosamine
GLUT: Gluteus maximus
H&E: Hematoxylin & eosin
IgG: Immunoglobulin G
LARGE1: Like-acetylglucosaminyltransferase 1
Large1^skm^: Skeletal muscle-specific deletion of *Large1*
LG: Laminin globular
LGMD: Limb girdle muscular dystrophy
Man: Mannose
MCK: Muscle creatine kinase
MGAT5B: Mannosyl α1,6-glycoprotein β1,6-*N-*acetyl-glucosaminyltransferase
NFH: Neurofilament-H
NMJ: Neuromuscular junction
PAX7: Paired box 7
POMGNT1: Protein *O*-linked mannose *N*-acetylglucosaminyltransferase 1
POMGNT2: Protein *O*-linked mannose *N*-acetylglucosaminyltransferase 2
POMK: Protein *O*-mannose kinase
POMT1: Protein *O*-mannosyltransferase 1
Pomt1^skm^: Skeletal muscle-specific deletion of *Pomt1*
POMT2: Protein *O*-mannosyltransferase 2
QUAD: Quadriceps
RXYLT1: Ribitol xylosyltransferase 1
TA: Tibialis anterior
TMEM5: Transmembrane protein 5
TVA: Transversus abdominis
WGA: Wheat-germ agglutinin
Xyl: Xylose
α-BTX: Alpha-bungarotoxin
α-DG: Alpha-dystroglycan
β-DG: Beta-dystroglycan
