# Supplementary material for: Sarcolemma resilience and skeletal muscle health require O-mannosylation of dystroglycan
Source: Skelet Muscle. 2025 Jan 9;15:1. doi: 10.1186/s13395-024-00370-2 (PMC11715199; doi:10.1186/s13395-024-00370-2)
Supplement: Supplementary file 1 — Supplementary Material 1. [file 13395_2024_370_MOESM1_ESM.pdf]

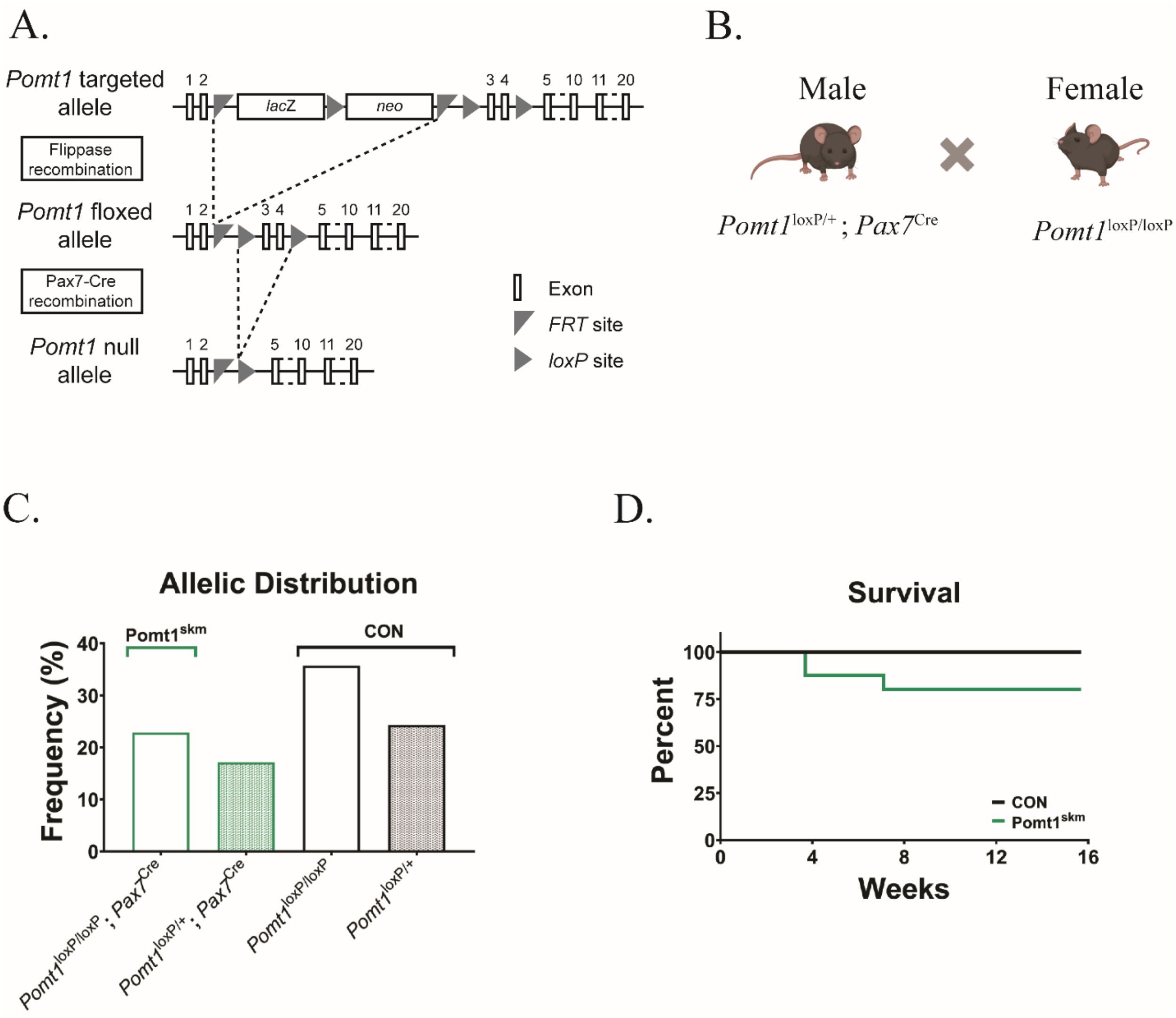

Supplemental Figure 1. **Pomt1^{skm} mouse line breeding and survival. A.** Design of the floxed *Pomt1* mouse line crossed with Cre under the *Pax7* promoter**. B.** Breeding scheme to generate *Pomt1*$^{loxP/loxP}$*; Pax7*^{Cre} (Pomt1^{skm}) mice using Cre-positive; heterozygous floxed males crossed with Cre-negative; homozygous floxed females. **C.** Allelic distribution of progeny from breeding strategy shown in B. *Pomt1*$^{loxP/loxP}$*; Pax7*^{Cre} represent the target mice (Pomt1^{skm}). Control (CON) mice included *Pomt1*$^{loxP/loxP}$ and *Pomt1*$^{loxP/+}$ genotypes. *Pomt1*$^{loxP/+}$*; Pax7*^{Cre} are included in the analysis but were not used as controls for other experiments. N = 70 mice total. **C.** Kaplan-Meier survival analysis for CON (*Pomt1*$^{loxP/loxP}$ and *Pomt1*$^{loxP/+}$) and Pomt1^{skm} mice. N = 16 per group.

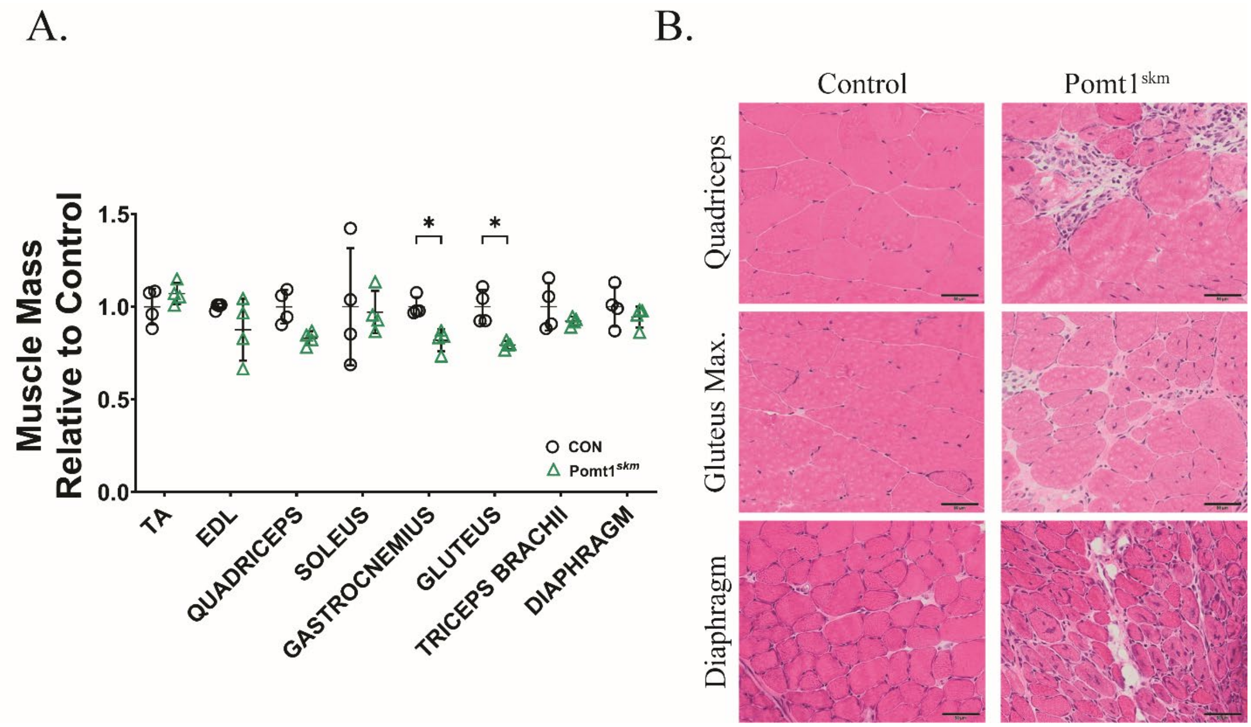

Supplemental Figure 2. **Skeletal muscle wet weights and histopathology. A.** Muscle mass in milligrams (mg) for different skeletal muscles from 13-week-old male Pomt1^{skm} and age- and gender-matched controls. Graph includes wet muscle mass relative to controls of the tibialis anterior (TA), extensor digitorum longus (EDL), quadriceps, soleus, gastrocnemius, gluteus maximus, triceps brachii, and diaphragm. N = 4 / group. Data expressed as mean ± standard deviation. P-values determined by unpaired t-test with Holm-Sidak post-hoc analysis. Gastrocnemius * = 0.0292; Gluteus * = 0.0324. **B.** Hematoxylin and eosin (H&E)-stained transverse cross-sections from quadriceps, gluteus maximus, and diaphragm muscles obtained from control or Pomt1^{skm} mice. Scale bar = 50 µm.

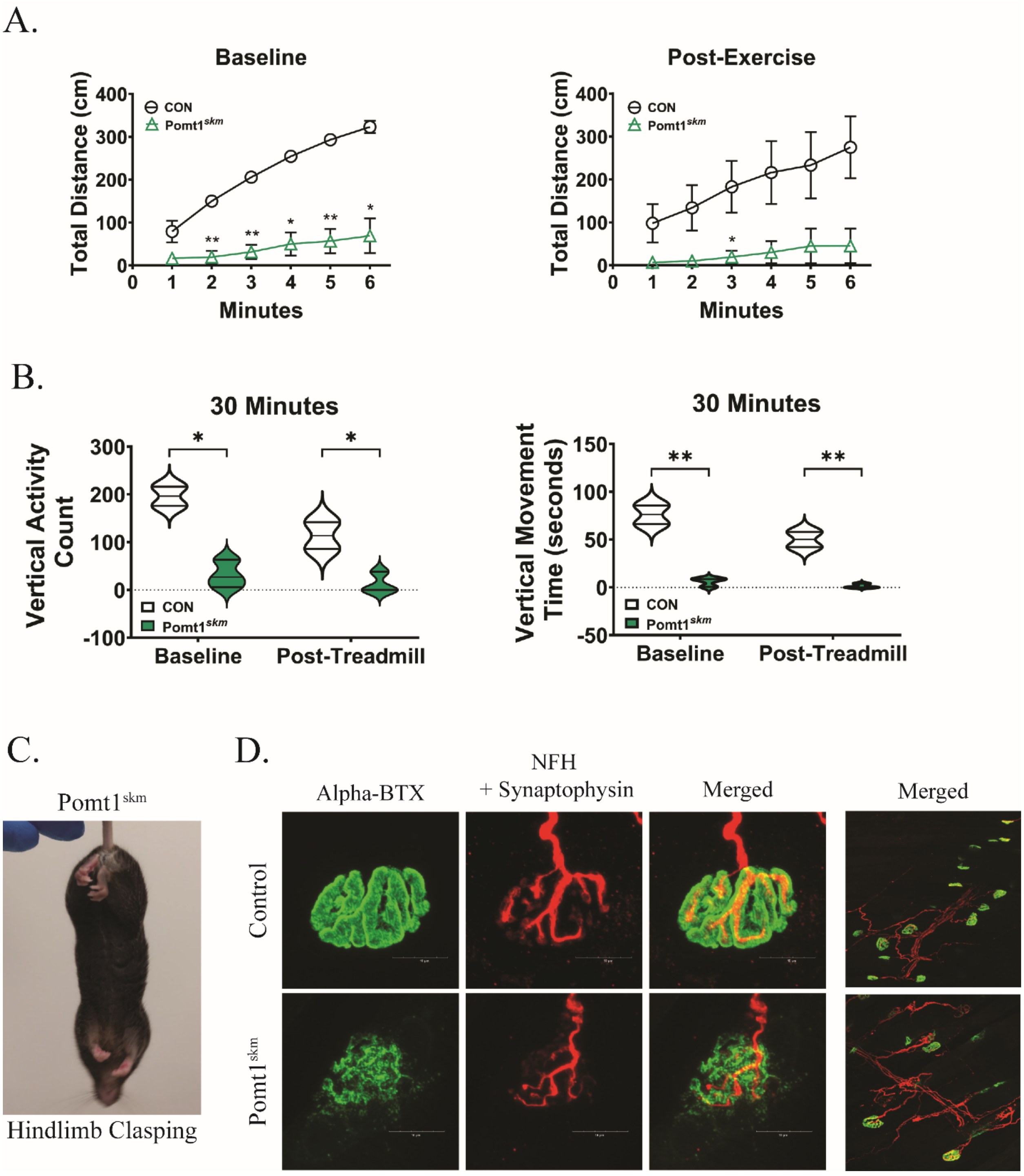
A.
Baseline
Post-Exercise
Total Distance (cm)
CON
Pomt1 skm
Minutes
B.
30 Minutes
Vertical Activity Count
Vertical Movement Time (seconds)
Baseline
Post-Treadmill
C.
Pomt1 skm
Hindlimb Clasping
D.
Alpha-BTX
NFH + Synaptophysin
Merged
Merged
Control
Pomt1 skm

Supplemental Figure 3. **Skeletal muscle deletion of *Pomt1* reduces voluntary activity and disrupts neuromuscular function. A-B.** Voluntary activity was assessed via open field activity assay. Mice were evaluated at baseline and immediately following a period of downhill treadmill running. **A.** Total distance traveled over the first 6 min in the open field chamber at baseline (*left panel*) and post-exercise (*right panel*). **B.** Vertical activity count (*left panel*) and movement time (*right panel*) over a 30 min period. For all statistical analyses, unpaired t-tests with Holm-Sidak post-hoc analysis were performed. Data expressed as mean ± standard deviation. * < 0.05; ** < 0.005. **C.** $Pomt1^{skm}$ mice display hindlimb clasping when held upside-down from their tail, a phenotype often associated with abnormal or inefficient muscle innervation. **D.** Immunofluorescence of neuromuscular junctions (NMJs) in whole mount transversus abdominis (TVA) muscles from 3- to 4-week-old control and $Pomt1^{skm}$ mice. Alpha-bungarotoxin (α-BTX) was used to detect post-synaptic acetylcholine receptors (AChRs). Anti-neurofilament H and anti-synaptophysin were used to detect motor neurons and the presynaptic terminal. Images were captured at 400X (first 3 columns) and 40X magnification (far right column).

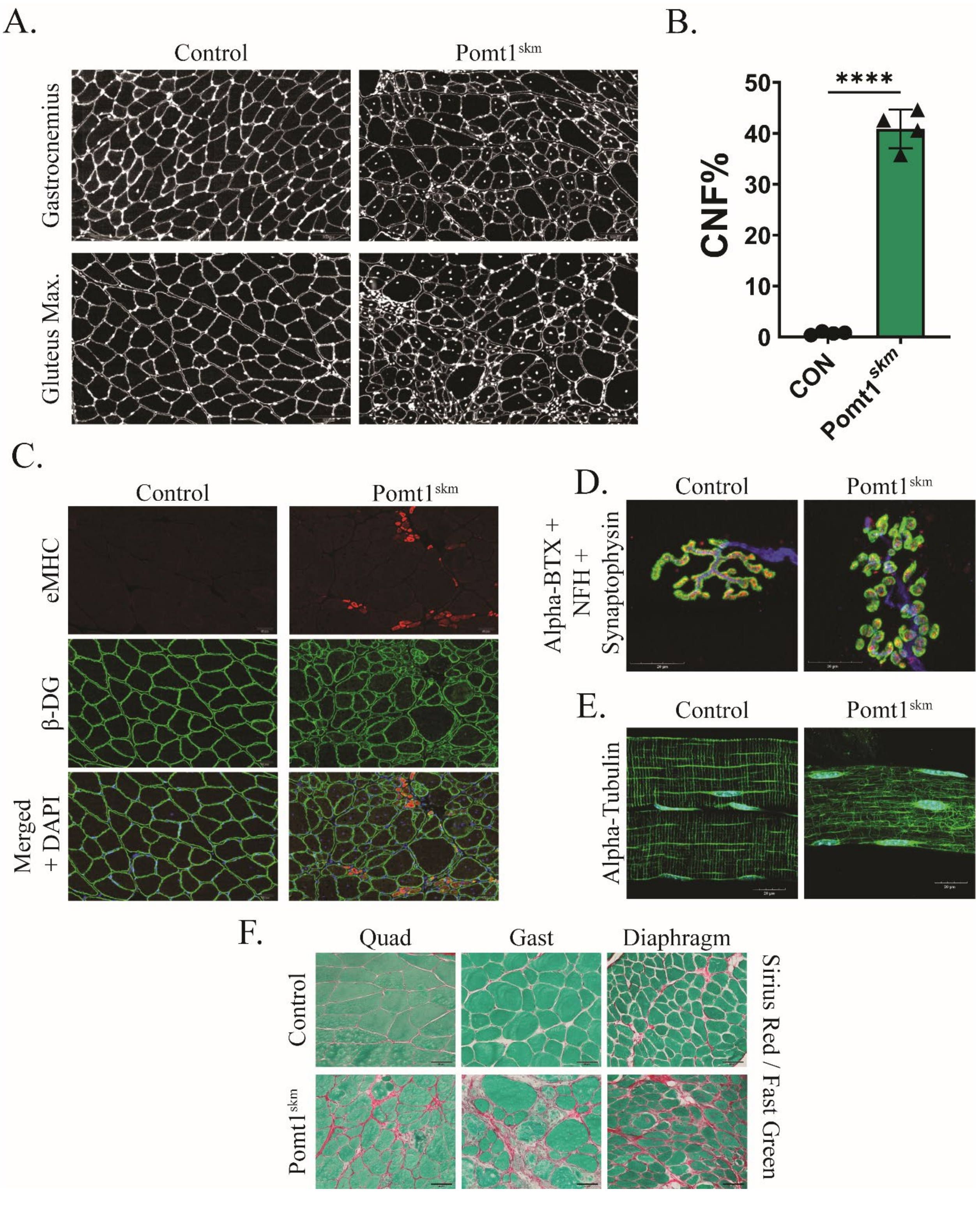
A.
Control
Pomt1skm
Gastrocnemius
Gluteus Max.
B.
CNF%
****
50
40
30
20
10
0
CON
Pomt1skm
C.
Control
Pomt1skm
eMHC
β-DG
Merged + DAPI
D.
Control
Pomt1skm
Alpha-BTX + NFH + Synaptophysin
E.
Control
Pomt1skm
Alpha-Tubulin
F.
Quad
Gast
Diaphragm
Control
Pomt1skm
Sirius Red / Fast Green

Supplemental Figure 4. **Deletion of *Pomt1* in skeletal muscle contributes to impaired muscle remodeling. A.** Immunofluorescence for β-DG and DAPI expression in gastrocnemius and gluteus maximus muscles from control and $Pomt1^{skm}$ mice to identify the localization of myonuclei within muscle fibers. Images captured at 10X magnification. Scale bar = 100 μm. **B.** Percentage of central nucleated fibers (CNF) from the gastrocnemius muscle of control and $Pomt1^{skm}$ mice. Data expressed as mean ± standard deviation. P-values determined by unpaired t-tests with Holm-Sidak post-hoc analysis. **** < 0.0001. **C.**, Immunofluorescence for embryonic myosin heavy chain (eMHC), β-DG, and DAPI in gluteus maximus muscles of control and $Pomt1^{skm}$ mice. **D.** Morphology of the neuromuscular junction to detect post-synaptic AChR clusters (α-BTX), neurons (NFH), and the pre-synapse (synaptophysin) in EDL muscles of control and $Pomt1^{skm}$ mice. **E.** Microtubule latticework detected via anti-alpha-tubulin in EDL muscles of control and $Pomt1^{skm}$ mice. **F.** Sirius red / fast green staining of quadriceps, gastrocnemius, and diaphragm muscles from control and $Pomt1^{skm}$ mice. Images captured at 10X magnification. Scale bar = 100 μm.

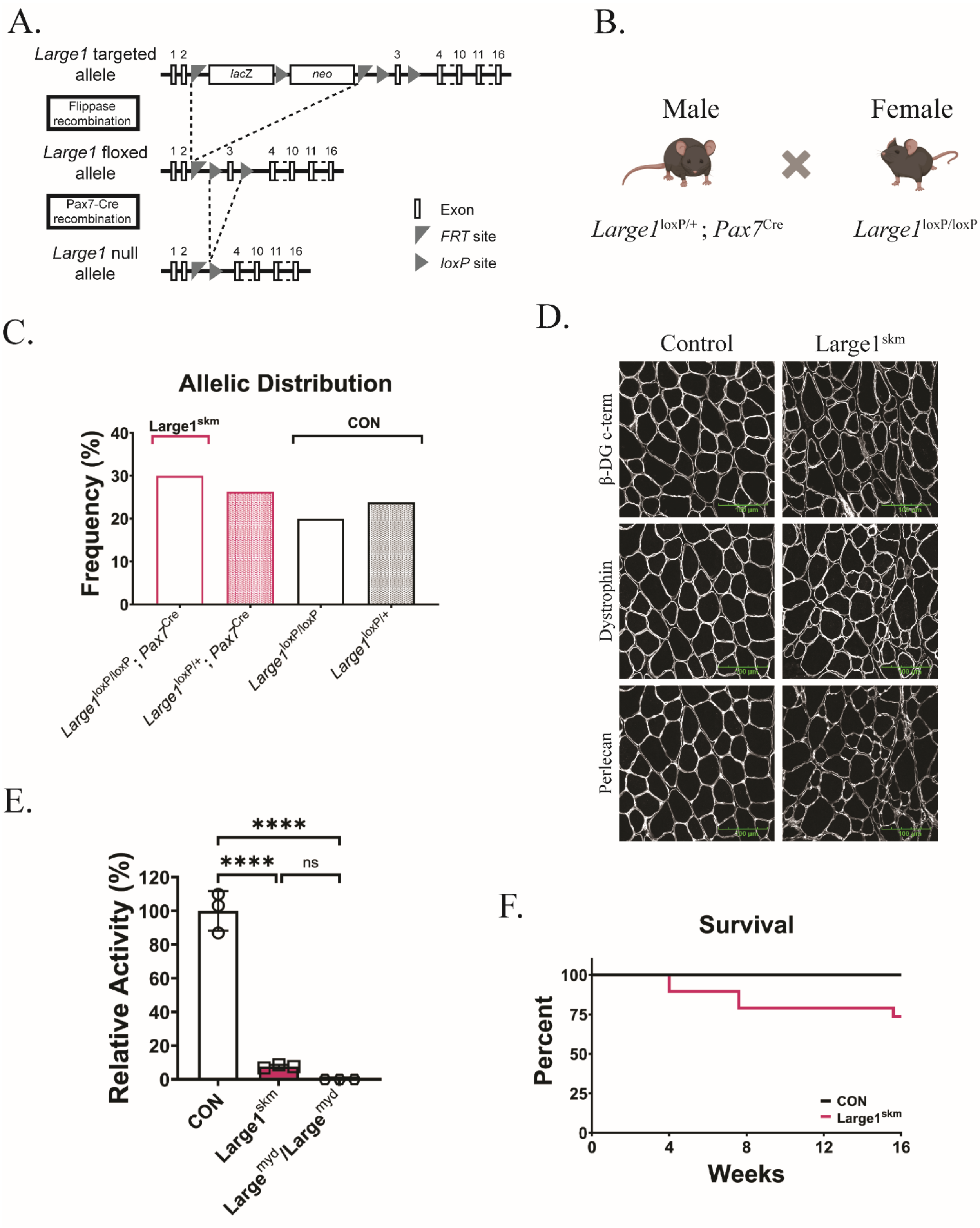
A.
Large1 targeted allele
1 2
lacZ
neo
3
4 10 11 16
Flippase recombination
Large1 floxed allele
Pax7-Cre recombination
Large1 null allele
Exon
FRT site
loxP site
B.
Male
Female
Large1loxP/+ ; Pax7Cre
Large1loxP/loxP
C.
Allelic Distribution
Large1skm
CON
Frequency (%)
0
10
20
30
40
Large1loxP/loxP ; Pax7Cre
Large1loxP/+ ; Pax7Cre
Large1loxP/loxP
Large1loxP/+
D.
Control
Large1skm
β-DG c-term
Dystrophin
Perlecan
E.
****
****
ns
Relative Activity (%)
0
20
40
60
80
100
120
CON
Large1skm
Largemyd/Largemyd
F.
Survival
Percent
0
25
50
75
100
0
4
8
12
16
Weeks
CON
Large1skm

Supplemental Figure 5. **Large1^{skm} mouse line generation and survival. A.** Design of the floxed *Large1* mouse line crossed with Cre under the *Pax7* promoter**. B.** Breeding scheme to generate *Large1*$^{loxP/loxP}$*; Pax7*^{Cre} (Large1^{skm}) mice using Cre-positive; heterozygous floxed males crossed with Cre-negative; homozygous floxed females. **C.** Allelic distribution of progeny from breeding strategy shown in B. *Large1*$^{loxP/loxP}$*; Pax7*^{Cre} represent the target mice (Large1^{skm}). Control (CON) mice included *Large1*$^{loxP/loxP}$ and *Large1*$^{loxP/+}$ genotypes. *Large1*$^{loxP/+}$*; Pax7*^{Cre} are included in the analysis but were not used as controls for other experiments. N = 80 mice total. **D.** Immunofluorescence for the c-terminus of β-DG (rabbit polyclonal AP83 antibody), dystrophin, and perlecan in transverse cross-sections of gastrocnemius muscles. Images shown at 40X magnification. Scale bar = 100 µm. **E.** Lysates from CON, Large1^{skm}, and *Large*^{myd}/*Large*^{myd} quadricep muscles from 21-week-old mice were assayed for LARGE enzyme activity. Relative activity (%) with respect to control. **** < 0.0001. **F.** Kaplan-Meier survival analysis for CON (*Large1* $^{loxP/loxP}$ and *Large1*$^{loxP/+}$) and Large1^{skm} mice. N = 13 CON and N = 19 Large1^{skm}.
